# Placental architectural characteristics following laser ablation within monochorionic twins complicated by twin–twin transfusion syndrome: A systematic review and meta‐analysis of outcomes

**DOI:** 10.1111/aogs.14891

**Published:** 2024-06-14

**Authors:** Jack Hamer, Nashwa Eltaweel, Rebecca Man, Matilde Rogerson, Victoria Hodgetts Morton, R. Katie Morris, Tamas Marton, Leo Gurney

**Affiliations:** ^1^ Institute of Applied Health Research University of Birmingham Birmingham UK; ^2^ Birmingham Women's and Children's NHS Foundation Trust Birmingham UK; ^3^ University Hospitals Coventry and Warwickshire NHS Trust Coventry UK; ^4^ Faculty of Medicine Semmelweis University Budapest Hungary

**Keywords:** abnormal cord insertion, anastomosis, artery–vein anastomosis, laser ablation, marginal cord, placenta, residual anastomoses, TTTS, twin–twin transfusion syndrome, velamentous cord

## Abstract

**Introduction:**

Twin–twin transfusion syndrome (TTTS) complicates approximately 10%–15% of all monochorionic twin pregnancies. The aim of this review was to evaluate the placental architectural characteristics within TTTS twins following laser and elucidate their impact on fetal outcomes and operative success.

**Material and Methods:**

Five databases were searched from inception to August 2023. Studies detailing post‐delivery placental analysis within TTTS twins post‐laser were included. Studies were categorized into two main groups: (1) residual anastomoses following laser and (2) abnormal cord insertion: either velamentous and/or marginal or proximate. The primary outcome was to determine the proportion of TTTS placentas with residual anastomoses and abnormal cord insertions post‐laser. Secondary outcomes included assessing residual anastomoses on post‐laser fetal outcomes and assessing the relationship between abnormal cord insertion and TTTS development. Study bias was critiqued using the Joanna Briggs Institute checklists and Cochrane risk of bias tool. Random‐effects meta‐analysis was used, and results were reported as pooled proportions or odds ratio (OR) with 95% confidence interval (CI). PROSPERO registration: CRD42023476875.

**Results:**

Twenty‐six studies, comprising 4013 monochorionic twins, were included for analysis. The proportion of TTTS placentas with residual anastomoses following laser was 24% (95% CI, 0.12–0.41), with a mean and standard deviation of 4.03 ± 2.95 anastomoses per placenta. Post‐laser residual anastomoses were significantly associated with intrauterine fetal death (OR, 2.38 [95% CI, 1.33–4.26]), neonatal death (OR, 3.37 [95% CI, 1.65–6.88]), recurrent TTTS (OR, 24.33 [95% CI, 6.64–89.12]), and twin anemia polycythemia sequence (OR, 13.54 [95% CI, 6.36–28.85]). Combined abnormal cord (velamentous and marginal), velamentous cord, and marginal cord insertions within one or both twins following laser were reported at rates of 49% (95% CI, 0.39–0.59), 27% (95% CI, 0.18–0.38), and 28% (95% CI, 0.21–0.36), respectively. Combined, velamentous and marginal cord insertions were not significantly associated with TTTS twins requiring laser (*p* = 0.72, *p* = 0.38, and *p* = 0.71, respectively) vs non‐TTTS monochorionic twins.

**Conclusions:**

To the best of our knowledge, this is the first review to conjointly explore outcomes of residual anastomoses and abnormal cord insertions within TTTS twins following laser. A large prospective study is necessitated to assess the relationship between abnormal cord insertion and residual anastomoses development post‐laser.

## Graphical abstract

Twins complicated by twin‐twin transfusion syndrome (TTTS) with residual anastomoses post‐laser ablation have a: 2‐fold increase of intrauterine fetal demise. 3‐fold increase of neonatal death. 14‐fold increase of post‐laser TAPS. 24‐fold increase of recurrent TTTS. Abnormal cord insertion (velamentous and/or marginal) does not influence the development TTTS within twins specifically undergoing laser ablation. Donor twins had a 5‐fold increase rate of abnormal cord insertion compared to recipient twins.

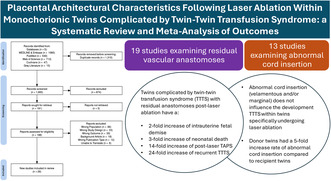

**Abbreviations**
AA: artery–artery
AV: artery–vein
CI: confidence intervals
IUFD: intrauterine fetal death
MC: monochorionic
NND: neonatal death
OR: odds ratios
sIUGR: selective intrauterine growth restriction
TAPS: twin anemia polycythemia sequence
TTTS: twin–twin transfusion syndrome
VV: vein–vein

**Key message**

The aim of this systematic review and meta‐analysis was to evaluate the placental architectural characteristics within monochorionic twins complicated by twin‐twin transfusion syndrome (TTTS) following laser ablation and elucidate their impact on fetal outcomes and operative success. To the best of our knowledge, this is the first review to conjointly explore outcomes of residual anastomoses and abnormal cord insertions within this twin cohort. This review enables clinicians the ability to clearly inform patients of potential post‐laser risks during pre‐and post‐operative counselling.

## 1 INTRODUCTION

Twin–twin transfusion syndrome (TTTS) is a significant complication that occurs in approximately 10%–15% of all monochorionic (MC) twin pregnancies.^1^ The pathophysiology for TTTS development involves the presence of vascular anastomoses on the chorionic surface, importantly unidirectional artery–vein (AV) anastomoses, which connect each fetal circulation across the MC placenta.^2^, ^3^, ^4^ A unidirectional imbalance of blood flow leads to characteristic changes in polyhydramnios in one twin (the recipient) and oligohydramnios in the other (the donor), which is pathognomic of TTTS. The severity of TTTS has been classified using the Quintero staging, with stages 2 and above recommended to undergo laser surgery.^5^, ^6^

Fetoscopic laser is an intrauterine treatment modality that intends to coagulate the intertwin vascular anastomoses.^7^, ^8^ The number of residual anastomoses post‐laser should be kept to a minimum, to reduce the risk of recurrent TTTS and post‐laser twin anemia polycythemia sequence (TAPS).^9^ Clinicians have aimed to achieve this by adapting the surgical technique from the selective to the Solomon method, ensuring to ablate the entire vascular equator from each placental edge. However, irrespective of surgical technique, the complete occlusion of anastomoses is not always achieved, as prior literature has documented at varying incidences upon placental examination.^10^, ^11^, ^12^, ^13^, ^14^, ^15^ There have been no previous systematic reviews describing or quantifying these residual anastomoses, or investigating factors that may influence numbers of residual anastomoses. The impact that residual anastomoses have on fetal outcomes also requires further investigation.

Abnormal placental cord insertion sites are more common within twin pregnancies compared to singletons.^16^ Specifically, velamentous cord insertion has been reported in approximately 2% of singletons, 7% of dichorionic twins, and between 12 and 36% of MC twins.^17^, ^18^, ^19^, ^20^ It is suspected that abnormal cord insertion may predispose to the development of TTTS within MC twins, although current published evidence is conflicting.^19^, ^21^, ^22^ There is a scarcity of available literature delineating the proportions of different types of abnormal cord insertion including how this influences the TTTS development for MC twins specifically requiring laser.

The aim of this systematic review and meta‐analysis is to evaluate the placental architectural characteristics within TTTS MC twins following laser ablation and elucidate their impact on fetal outcomes and operative success.

## 2 MATERIAL AND METHODS

This review was prospectively registered with PROSPERO (CRD42023476875) and results have been reported in accordance with the preferred reporting of systematic reviews and meta‐analysis (PRISMA) guidelines.^23^ MEDLINE, EMBASE, Cochrane, Web of Science, and PubMed databases were searched from inception to August 2023. The MEDLINE/EMBASE search is detailed in Table S1. Search terms and functions were appropriately adapted for each database. No language restrictions were applied.

Rayyan software was acquired to manage title, abstract, and full manuscript screening, including subsequent data extraction. All abstracts and full texts were reviewed by two authors (JH and NE) and conflicts were discussed with a third author (LG). Reference lists were additionally screened to identify additional gray literature citations eligible for inclusion. Studies specifically detailing post‐delivery placental analysis within MC twins complicated by TTTS that underwent intrauterine laser ablation were included. Pathology placental analysis was required to be detailed within the study methodological section for inclusion to ensure true estimates of placental anatomical characteristics could be established. In the uncommon event that an interventional group included patients undergoing various interventions (laser ablation or amnio drainage), then studies were deemed eligible for inclusion if a minimum of 60% of the cohort had undergone laser ablation. This was to capture a larger cohort of included studies while ensuring the results following laser ablation were not excessively diluted by alternative interventions. Later subgroup analysis within the results excluded these studies to determine their influence on results. Studies examining singleton, dichorionic, monoamniotic, and higher‐order pregnancies were excluded. Included studies were categorized into two main groups: (1) studies identifying the presence of residual vascular anastomoses and (2) studies describing abnormal cord insertion: either velamentous and/or marginal or proximate. Several included studies encompassed both categories.

### 2.1 Data analysis

Outcomes analyzed were individualized based on each group of included studies and were predetermined by an initial scoping review and prior to data extraction. For studies examining residual vascular anastomoses, the primary outcome was to determine the proportion of placentas with residual anastomoses present following laser ablation. Further analysis determines the mean number of residual anastomoses, subtypes of residual anastomoses, and their diameters. Secondary outcomes included the impact of placental position during laser on the number of placentas with residual anastomoses. Further analyses assessed the impact of residual anastomoses on single and/or double intrauterine fetal death (IUFD), neonatal death (NND), recurrent TTTS, and post‐laser TAPS.

For studies examining abnormal cord insertion, the primary outcome was to determine the proportion of different types of abnormal cord insertion within the TTTS cohort undergoing laser ablation. Secondary outcomes included the influence of abnormal cord insertion on the development of TTTS vs other MC cohorts and the impact of abnormal cord insertion on IUFD and NND. Outcomes were considered eligible for meta‐analysis if three or more studies were suitable for inclusion in the analysis. This was intended to improve the reliability of the effect estimates, given the methodological heterogeneity within the available literature.^24^

Predetermined subgroup analysis was individualized for each group. For studies examining number of placentas with residual anastomoses, this included: dual survivors and single/double IUFD, Quintero staging, and laser ablation technique. For studies examining abnormal cord insertion, this included: donor/recipient cord insertion and studies with or without the total cohort undergoing laser.

Review Manager 5.4 (RevMan) was used to meta‐analyze pairwise data.^25^ Dichotomous outcome data were assessed with 2 × 2 tables constructed to calculate odds ratios (OR) and 95% confidence intervals (CI). Mantel–Haenszel method random‐effects models were utilized for meta‐analysis. Summary OR were configured with forest plots, allowing visual inspection and quantitative assessment for heterogeneity using *I* ^2^.^26^ Zero‐cell adjustments to 0.5 were used as required. Studies containing raw data individually reporting on abnormal cord insertion and residual anastomoses were combined to form a mean and standard deviation (SD). Continuous variables, such as residual anastomoses and vascular subtypes, reported with an overall median and range were translated to proxy mean and SD using methodological equations described by Hozo et al and Wan et al.^27^, ^28^ Calculated means and SD for relevant groups from each study were combined together for an overall mean and SD using the Cochrane method for combing means.^29^

Proportional data and 95% CI were calculated using R version 4.3.0.^30^ Random effects models were used to meta‐analyze estimates of the overall proportional data due to the high likelihood of statistical heterogeneity. Heterogeneity arising from methodological diversity between studies, particularly due to nuances in placental analysis techniques and reporting, as well as specific inclusion/exclusion study, led to a variation in observed intervention effects between studies. The inverse variance method with logit transformation of the rate was used. Summary proportions and CI were configured with forest plots, including quantitative assessment for heterogeneity using *I* ^2^. *I* ^2^ values below 25% were considered low, approximately 50% considered moderate, and above 75% considered high levels of heterogeneity.^26^ Subgroup proportional data analysis was configured using visual forest plots, using random effects testing to assess for subgroup statistical differences with *p* values.

### 2.2 Risk of bias and trustworthiness

Risk of bias in observational studies was assessed by two reviewers (JH and NE) using the most relevant and recent critical appraisal checklists from the Joanna Briggs Institute.^31^ The Cochrane risk of bias tool (RoB2) was used to assess bias in interventional studies.^32^ Any discrepancies with bias assessment were arbitrated with a third author (LG).

## 3 RESULTS

**FIGURE 1:**
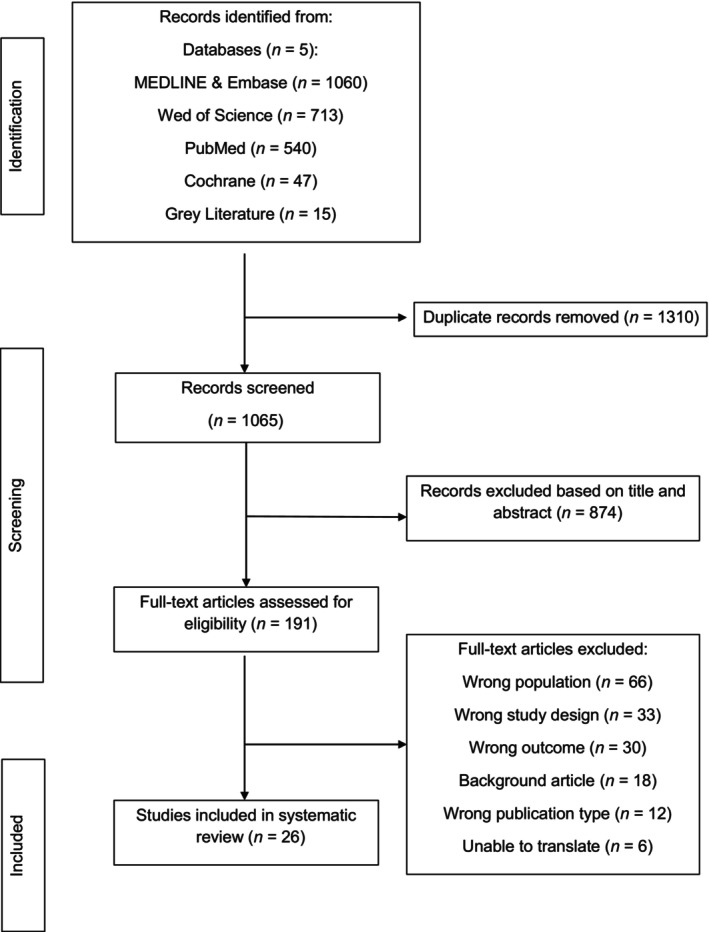
PRISMA flowchart summarizing inclusion of studies in systematic review and meta‐analysis.

The database searches identified 2360 abstracts. Fifteen citations were identified through gray literature searches. A total of 1310 duplicate abstracts were removed, leaving 1065 abstracts for screening. A total of 191 full texts were reviewed, from which 26 papers, comprising 4013 MC twins were eligible for inclusion.^4^, ^9^, ^11^, ^12^, ^15^, ^19^, ^20^, ^33^, ^34^, ^35^, ^36^, ^37^, ^38^, ^39^, ^40^, ^41^, ^42^, ^43^, ^44^, ^45^, ^46^, ^47^, ^48^, ^49^, ^50^ The PRISMA flowchart details this below (Figure 1). The characteristics of the included studies are presented in Table S2. The most common reason for exclusion during the full‐text screening was due to reporting of the wrong population (*n* = 66). This commonly referred to MC twins uncomplicated by TTTS; or twins complicated by TTTS that did not undergo laser ablation. Studies excluded following full‐text screening are presented in Table S3. All papers included were written in English. For six studies it was not possible to obtain translation, therefore, these were excluded from analysis.^51^, ^52^, ^53^, ^54^, ^55^, ^56^

### 3.1 Residual anastomoses following laser ablation

Nineteen included studies presented data on residual anastomoses following laser ablation within MC twins complicated by TTTS.^4^, ^10^, ^11^, ^12^, ^13^, ^14^, ^15^, ^33^, ^34^, ^36^, ^37^, ^38^, ^39^, ^41^, ^42^, ^44^, ^45^, ^47^, ^48^, ^50^ One study was an RCT, two were prospective cohort studies, seven were retrospective cohort studies, and nine were case series papers.

#### 3.1.1 Primary outcome

**FIGURE 2:**
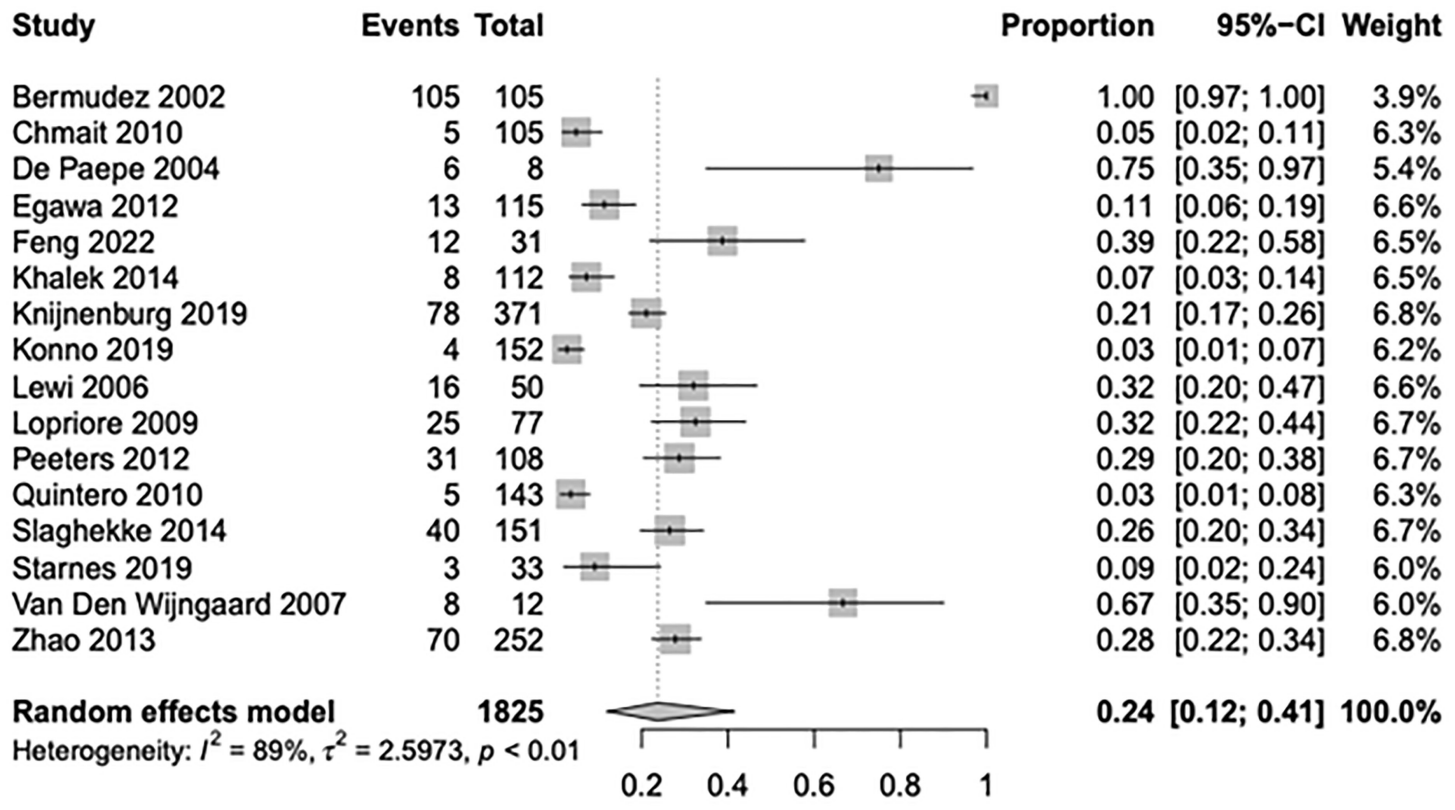
Individual study proportion rates for overall residual anastomoses following laser ablation within twin–twin transfusion monochorionic twins.

Sixteen studies (1825 placentas) specifically reported on the proportion of placentas with residual vascular anastomoses following laser for TTTS twins. Residual anastomoses were found in 24% ([95% CI, 0.12–0.41]; *I* ^2^ = 89%) (Figure 2) of post‐laser placentas, with a mean number and SD of 4.03 ± 2.95 anastomoses present (8 studies, 213 placentas) (Table 1).

**TABLE 1:** Studies examining the means and diameters of residual anastomoses, including subtypes for twin–twin transfusion monochorionic twins following laser ablation.

Paper	Participants (*n*)	Mean number ± standard deviation (SD)
Overall mean numbers of residual anastomoses present per placenta		
Bermudez (2002)^33^	105	4.76 ± 2.48
Chmait (2010)^34^	5	1.40 ± 0.49
De Paepe (2004)^36^	6	3.50 ± 1.89
Khalek (2014)^41^	8	2.88 ± 2.20
Lewi (2006)^42^	16	1.69 ± 0.68
Lopriore (2009)^44^	25	2.50 ± 1.02
Slaghekke (2014)^47^	40	4.65 ± 4.57
Van Den Wijngaard (2007)^15^	8	3.88 ± 2.62
Total	213	4.03 ± 2.95

Paper	Participants (*n*)	Mean number ± SD	Participants (*n*)	Mean diameter ± SD
Overall mean numbers of AA residual anastomoses present per placenta with residual anastomoses				
De Paepe (2004)^36^	2	1.00 ± 0.00		
Feng (2022)^10^			7	2.23 ± 1.33
Khalek (2014)^41^	2	1.50 ± 0.50		
Lewi (2006)^42^	2	1.00 ± 0.00	2	1.90 ± 0.70
Van Den Wijngaard (2007)^15^	3	1.00 ± 0.00		
Wang (2022)^4^	7	1.00 ± 0.00	7	3.25 ± 1.61
Total	16	1.06 ± 0.21	16	2.64 ± 1.45
Overall mean numbers of AV residual anastomoses present per placenta with residual anastomoses				
De Paepe (2004)^36^	5	3.60 ± 1.36		
Feng (2022)^10^			2	1.30 ± 0.71
Khalek (2014)^41^	1	1.00 ± 0.00		
Lewi (2006)^42^	14	1.07 ± 0.26	14	1.22 ± 0.58
Van Den Wijngaard (2007)^15^	7	2.71 ± 2.19		
Wang (2022)^4^	9	4.50 ± 2.68	9	3.18 ± 2.11
Total	36	2.60 ± 2.19	25	1.93 ± 1.61
Overall mean numbers of VV residual anastomoses present per placenta with residual anastomoses				
De Paepe (2004)^36^	1	1.00 ± 0.00		
Feng (2022)^10^			4	1.70 ± 0.54
Khalek (2014)^41^	3	1.66 ± 0.94		
Lewi (2006)^42^	3	1.00 ± 0.00	3	1.60 ± 0.75
Van Den Wijngaard (2007)^15^	7	1.13 ± 0.35		
Wang (2022)^4^	9	1.00 ± 0.00	9	3.28 ± 1.91
Total	23	1.13 ± 0.40	16	2.57 ± 1.66
Overall mean numbers of VA residual anastomoses present per placenta with residual anastomoses				
Feng (2022)^10^			5	1.46 ± 0.62
Khalek (2014)^41^	6	2.30 ± 0.94		
Lewi (2006)^42^	7	1.00 ± 0.00	7	0.73 ± 0.28
Van Den Wijngaard (2007)^15^	1	1.00 ± 0.00		
Total	14	1.57 ± 0.90	12	1.03 ± 0.57

*Note*: Only the first author is given for each study. Filled color means information is not available for that cell.

Abbreviations: AA, artery–artery; AV, artery–vein; VA, vein–artery; VV, vein–vein.

**FIGURE 3:**
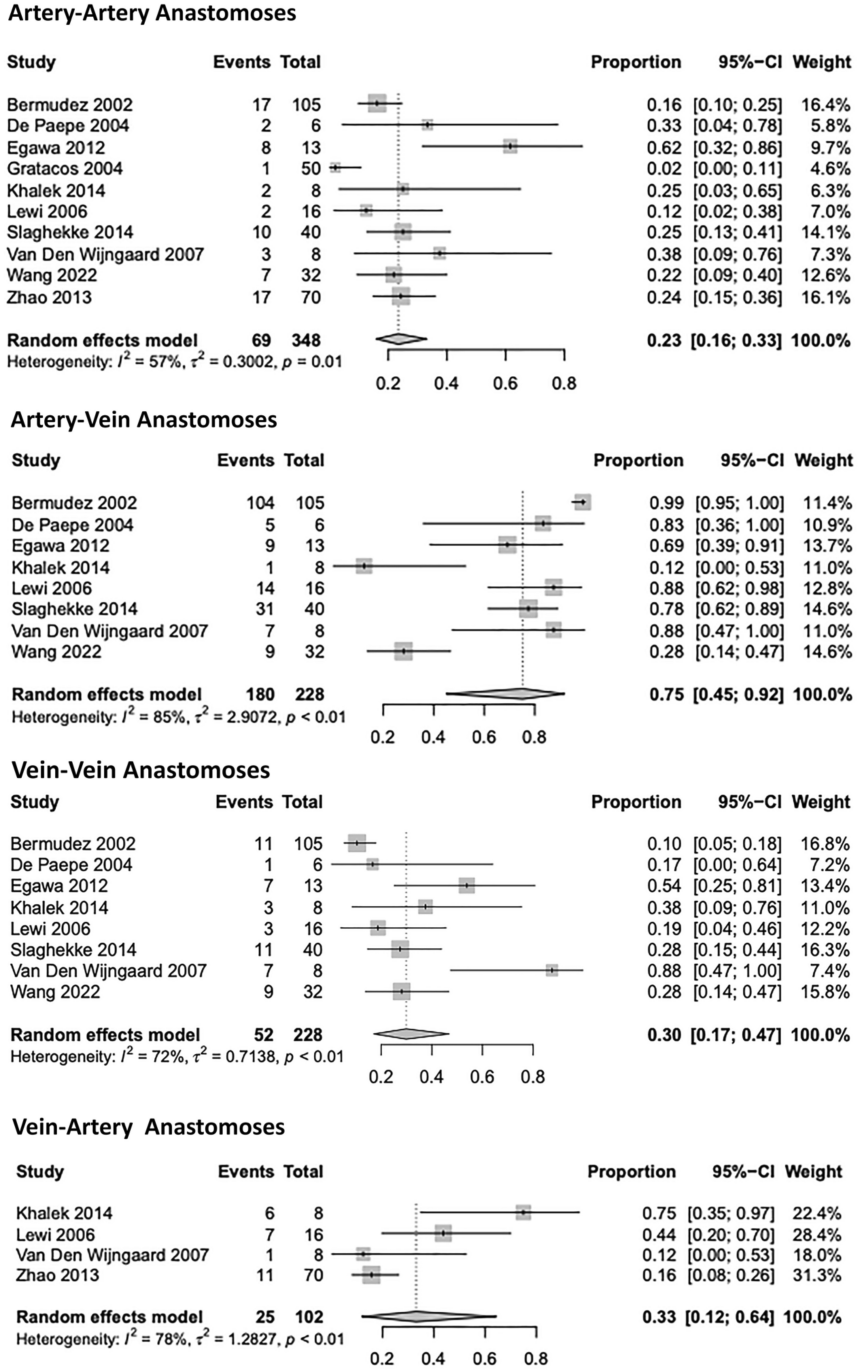
Individual study proportion rates for each residual anastomosis subtype following laser ablation within twin–twin transfusion monochorionic twins.

The overall proportions of each anastomosis subtype were determined from placentas only demonstrating residual anastomoses post‐laser. Mean subtype anastomosis numbers and diameters were determined from placentas only demonstrating the specific anastomosis subtype in question. Ten studies (348 placentas) demonstrated an overall artery–artery (AA) proportion of 23% ((95% CI, 0.16–0.33); *I* ^2=^57%), with a mean number and SD of 1.06 ± 0.21 anastomoses present (five studies, 16 placentas). The mean diameter and SD of AA residual anastomoses was 2.64 mm ± 1.45 (3 studies, 16 placentas). Eight studies (228 placentas) demonstrated an overall AV proportion of 75% ((95% CI, 0.45–0.92); *I* ^2=^85%), with a mean number and SD of 2.60 ± 2.19 anastomoses present (6 studies, 36 placentas). The mean diameter and SD of AV residual anastomoses was 1.93 mm ± 1.61 (3 studies, 25 placentas). Eight studies (228 placentas) demonstrated an overall vein–vein (VV) proportion of 30% ((95% CI, 0.17–0.47); *I* ^2=^72%), with a mean number and SD of 1.13 ± 0.40 anastomoses present (5 studies, 23 placentas). The mean diameter and SD of VV residual anastomoses was 2.57 mm ± 1.66 (3 studies, 16 placentas). Four studies (102 placentas) additionally evaluated the presence of vein–artery anastomoses on placental examination; a unidirectional anastomosis comparative to an AV anastomosis, however, with an opposite directional flow. Vein–artery (VA) anastomoses demonstrated an overall proportion of 33% ((95% CI, 0.12–0.64); *I* ^2=^78%), with a mean number and SD of 1.57 ± 0.90 anastomoses present (3 studies, 14 placentas). The mean diameter and SD of VA residual anastomoses was 1.03 mm ± 0.57 (2 studies, 12 placentas). Proportions are visually represented in Figure 3. Results of overall and subtype mean numbers and diameters for each study are presented in Table 1.

#### 3.1.2 Secondary outcomes

The OR for all presented outcomes from each included study are demonstrated in Tables 2 and 3. Five studies examined the rate of IUFD for TTTS MC twins following laser ablation. The presence of residual anastomoses posed a significantly greater risk of single or double IUFD for MC twins (OR, 2.38 (95% CI, 1.33–4.26); *I* ^2=^60%). Three studies demonstrated that the rate of NND for TTTS MC twins was significantly greater for twins with residual anastomoses following laser ablation (OR, 3.37 (95% CI, 1.65–6.88); *I* ^2=^0%).

**TABLE 2:** Studies examining residual anastomoses following laser ablation for twin–twin transfusion monochorionic twins on the impact of different fetal outcomes.

Paper	Events out of total participants with residual anastomoses (*n*/*N*)	Events out of total participants without residual anastomoses (*n*/*N*)	OR effect estimate (95% CI)	*I* ^2^
Rate of intrauterine fetal demise (single or dual)				
De Paepe (2004)^36^	1/5	1/2	0.25 (0.01–8.56)	
Egawa (2012)^37^	3/13	4/102	7.35 (1.44–37.60)	
Knijnenburg (2019)^11^	10/78	24/293	1.65 (0.75–3.61)	
Konno (2019)^12^	0/4	34/148	0.37 (0.02–7.02)	
Lewi (2006)^42^	7/16	0/34	54.47 (2.85–1042.24)	
Total	116	579	2.38 (1.33–4.26)	60%
Neonatal death				
De Paepe (2004)^36^	1/8	0/2	1.00 (0.03–33.32)	
Egawa (2012)^37^	0/20	2/196	1.90 (0.09–40.89)	
Knijnenburg (2019)^11^	14/136	16/538	3.74 (1.78–7.88)	
Total	164	736	3.37 (1.65–6.88)	0%
Post‐Laser TAPS				
Chmait (2010)^34^	1/5	0/100	67.00 (2.38–1885.08)	
Feng (2022)^10^	7/19	2/21	5.54 (0.98–31.25)	
Khalek (2014)^41^	0/8	1/104	4.06 (0.15–107.45)	
Lopriore (2009)^44^	11/25	0/52	83.28 (4.63–1499.17)	
Slaghekke (2014)^47^	19/40	1/111	99.52 (12.63–784.26)	
Van Den Wijngaard (2007)^15^	0/12	1/14	0.36 (0.01–9.68)	
Zhao (2013)^50^	1/4	35/248	2.03 (0.21–20.06)	
Total	114	650	13.54 (6.36–28.85)	60%
Recurrent TTTS				
Chmait (2010)^34^	0/5	1/100	6.03 (0.22–165.76)	
Egawa (2012)^37^	4/13	0/102	97.11 (4.85–1943.40)	
Lewi (2006)^42^	3/16	0/34	17.89 (0.86–369.97)	
Slaghekke (2014)^47^	5/40	0/111	34.55 (1.86–640.33)	
Zhao (2013)^50^	0/4	5/248	4.92 (0.23–102.99)	
Total	78	595	24.33 (6.64–89.12)	0%

*Note*: Only the first author is given for each study. Filled color means data entry is not applicable.

Abbreviations: CI, confidence interval; OR, odds ratio; TAPS, twin anemia polycythemia sequence; TTTS, twin–twin transfusion syndrome.

**TABLE 3:** Studies examining placental position and Quintero staging for twin–twin transfusion monochorionic twins and the influence on residual anastomoses post‐laser.

Paper	Cases of residual anastomoses with anterior placental position out of total cases (*n*/*N*)	Cases of residual anastomoses with posterior placental position out of total cases (*n*/*N*)	OR effect estimate (95% CI)	*I* ^2^
Chmait (2010)^34^	2/42	3/63	1.00 (0.16–6.25)	
Knijnenburg 2019	38/156	40/215	1.41 (0.85–2.33)	
Lewi (2006)^42^	8/24	8/26	1.13 (0.34–3.69)	
Lopriore (2009)^44^	10/29	15/48	1.16 (0.43–3.08)	
Peeters (2012)^45^	13/45	18/63	1.02 (0.44–2.36)	
Quintero (2010)^13^	3/70	2/73	1.59 (0.26–9.81)	
Total	366	488	1.26 (0.88–1.80)	0%

Paper	Cases of residual anastomoses with quintero stage I/II out of total cases (*n*/*N*)	Cases of residual anastomoses with quintero stage III/IV out of total cases (*n*/*N*)	OR effect estimate (95% CI)	*I* ^2^
Chmait (2010)^34^	0/36	5/69	0.16 (0.01–2.99)	
De Paepe (2004)^36^	1/1	4/5	1.00 (0.02–40.28)	
Knijnenburg 2019	37/171	41/200	1.07 (0.65–1.77)	
Total	208	274	0.97 (0.60–1.57)	0%

*Note*: Only the first author is given for each study. Filled color means data entry is not applicable.

Abbreviations: CI, confidence interval; OR, odds ratio.

Residual anastomoses following laser ablation significantly increased the risk of post‐laser TAPS (OR, 13.54 (95% CI, 6.36–28.85); *I* ^2=^60%) (7 studies, 763 placentas) and the recurrence of post‐laser TTTS (OR, 24.33 (95% CI, 6.64–89.12); *I* ^2=^0%) (5 studies, 595 placentas).

Placental positioning intraoperatively and its relation to residual anastomoses following laser was discussed by several authors. Six papers (488 placentas) compared anterior vs posterior placental positioning and did not demonstrate a significant difference in the proportion of placentas with residual anastomoses (OR, 1.26 (95% CI, 0.88–1.80); *I* ^2^ = 0%) (Table 3).

#### 3.1.3 Subgroup analysis

**FIGURE 4:**
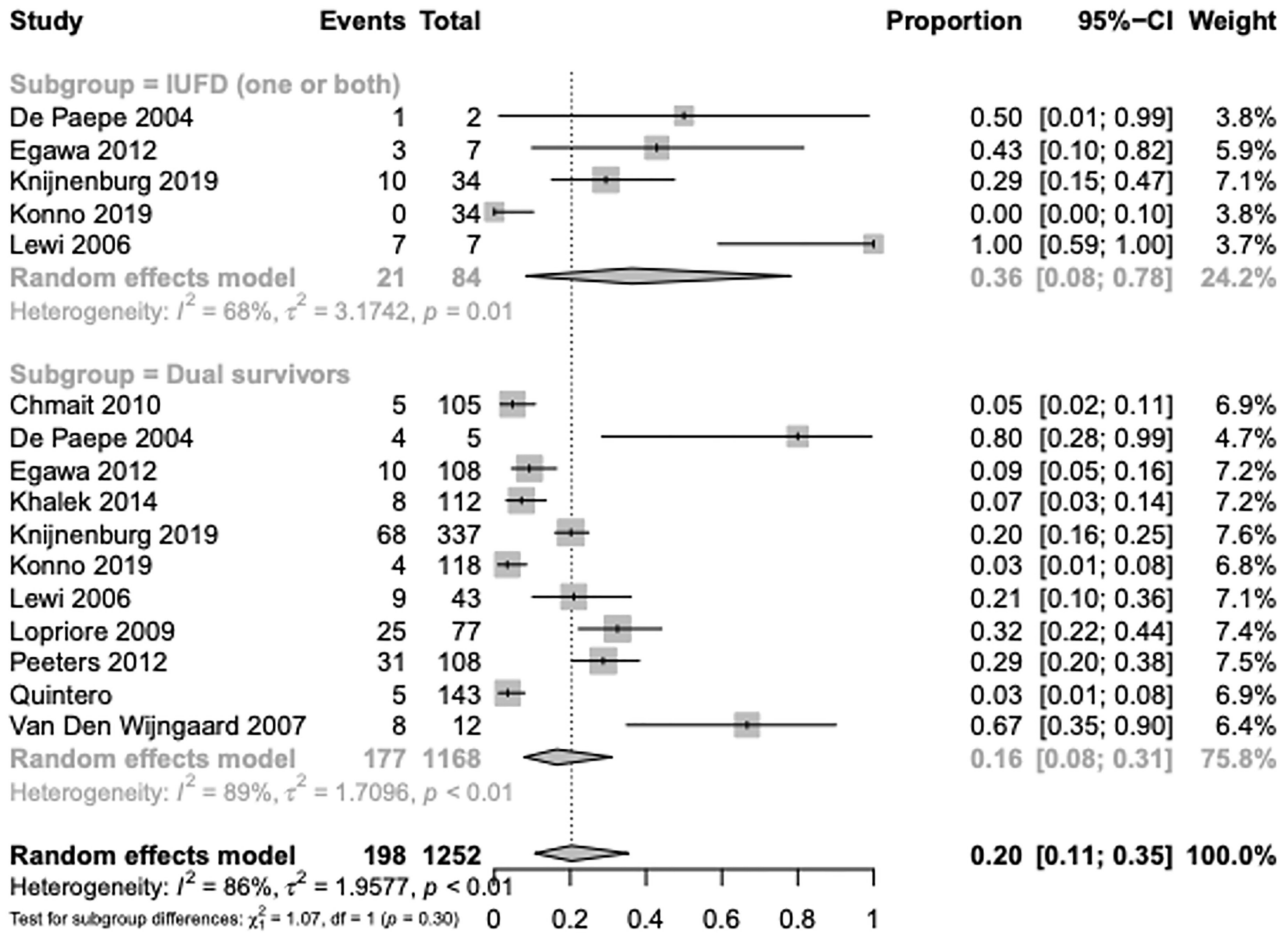
Individual study proportion rates for overall residual anastomoses following laser ablation within cases of intrauterine fetal demise (single or double) and dual survivors for twin–twin transfusion syndrome monochorionic twins.

Several studies only included placental analyses from dual survivors, due to the potential for placental maceration following single or double IUFD, which could impede assessment of residual anastomoses. For studies explicitly commenting on the inclusion of either dual survivors, or both survivors and IUFD (single or double), while also specifying the rates of residual anastomoses for each group, the proportional data were calculated. The proportion of placentas with residual anastomoses within dual survivors was 16% (95% CI, 0.08–0.31; *I* ^2^ = 89%, 11 studies) vs 36% (95% CI, 0.08–0.78; *I* ^2^ = 68%, 5 studies) for studies with single or double IUFD. Analysis of subgroup differences between survivors and IUFD was not significant (*p* = 0.30) (Figure 4). Subgroup difference analysis specifically for AV anastomoses could not be performed as included studies were too few.

Three studies detailed the number of residual anastomoses for each Quintero stage. Quintero stages 1 and 2 were combined and compared against combined stages 3 and 4 to enable meta‐analysis. There was no statistical difference in the number of residual anastomoses following laser ablation between Quintero stages (OR, 0.97 (95% CI, 0.60–1.57); *I* ^2=^0%) (Table 3).

Subgroup analysis of residual anastomoses for Solomon vs selective laser technique could not be performed. Many studies within this review did not specify the laser technique used. Consequently, only two studies were within the Solomon group, including one RCT, hindering meta‐analysis of surgical technique.^11^, ^47^

#### 3.1.4 Risk of bias

The full risk of bias assessments for all included studies is detailed in Table S2, within the Supporting Information. Sixteen studies were graded as low risk, two studies as moderate risk, and one study as high risk of bias. The included RCT was low risk across all domains of the Cochrane RoB2 tool. The most common reasons for moderate‐ and high‐risk scores were due to unclear reporting of inclusion criteria and no clear exclusion criteria mentioned.

### 3.2 Abnormal cord insertion

Thirteen studies were eligible for inclusion in the analysis of abnormal cord insertion within TTTS MC twins undergoing laser ablation.^4^, ^19^, ^20^, ^34^, ^35^, ^38^, ^40^, ^43^, ^46^, ^47^, ^48^, ^49^, ^50^ Abnormal cord insertions encompassed velamentous, marginal, and proximate cord insertions. One study was an RCT, one was a prospective case series, two studies were retrospective case series, two were prospective cohort studies, and seven were retrospective cohort studies. The characteristics are included in Table S2, within the Supporting Information.

#### 3.2.1 Primary outcome

**FIGURE 5:**
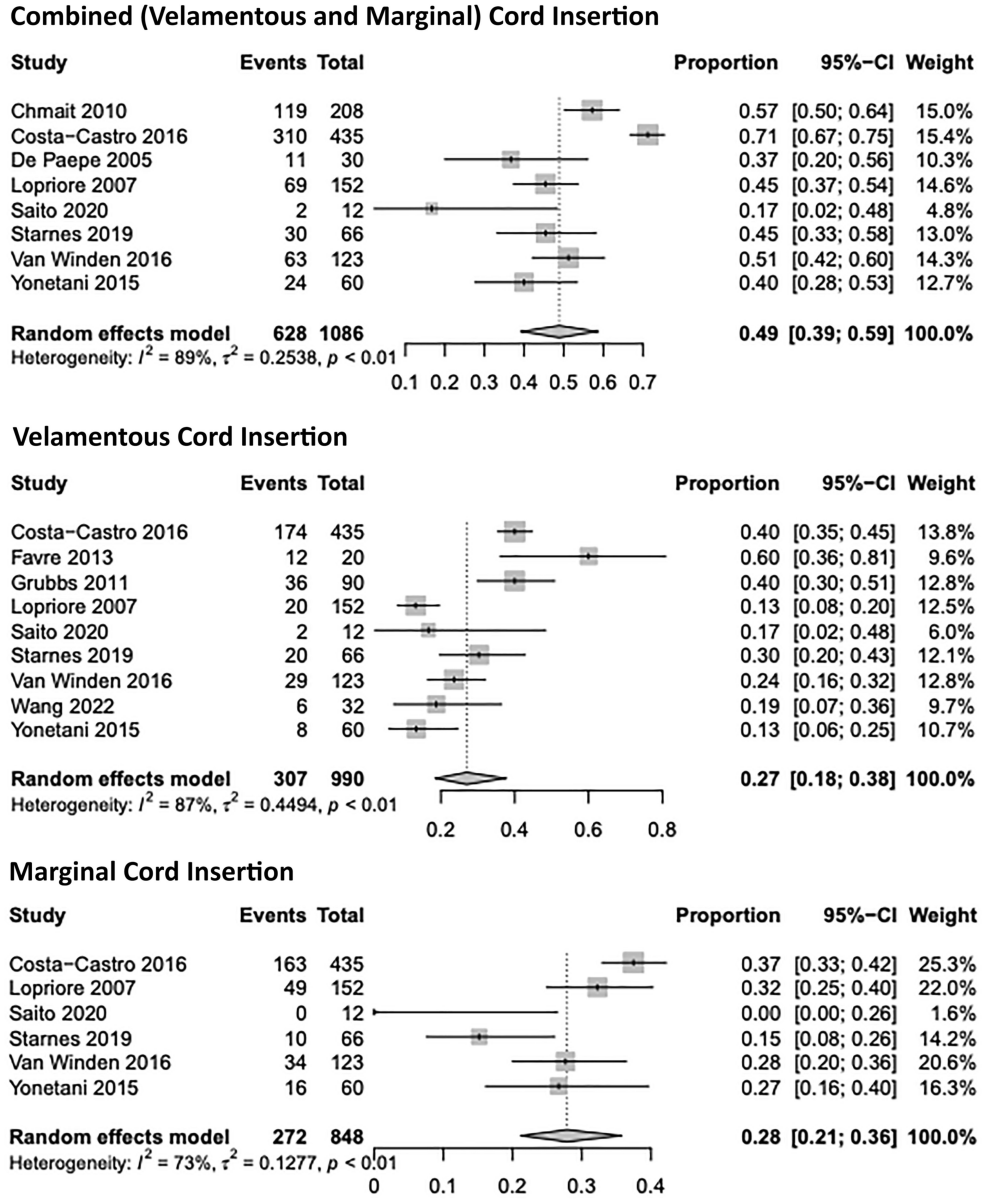
Individual study proportion rates for abnormal cord insertion (combined, velamentous, and marginal) within twin–twin transfusion monochorionic twins following laser ablation.

Eight studies specifically explored the proportion of combined velamentous and marginal cord insertion in either one or both TTTS MC twins that underwent laser. Overall combined abnormal cord insertion (velamentous and marginal) was reported at a rate of 49% ((95% CI, 0.39–0.59); *I* ^2^ = 89%) for one or both TTTS twins upon placental examination following laser. Overall velamentous insertion was reported within nine studies at a rate of 27% ((95% CI, 0.18–0.38); *I* ^2^ = 87%) in post‐laser placentas. Overall marginal cord insertion was reported within six studies at a rate of 28% ((95% CI, 0.21–0.36); *I* ^2^ = 73%) for one or both twins. Two included studies reported the rate of proximate cord insertion within one or both twins. The number of studies was insufficient to meta‐analyze; however, rates were both similar at approximately 1%. The individual proportions can be visualized in Figure 5.

#### 3.2.2 Secondary outcome

The OR for all presented outcomes from each included study are demonstrated in Table 4. The rates of abnormal cord insertion within TTTS MC twins undergoing laser ablation were compared to several other twin groups within the included papers. Comparison groups included MC twins uncomplicated by TTTS, MC twins complicated by TTTS not undergoing laser, and MC twins complicated with both TTTS and selective intrauterine growth restriction (sIUGR). Studies comparing abnormal cord insertion in MC twins with TTTS undergoing laser ablation vs MC twins uncomplicated by TTTS were the only group containing the required number of studies for meta‐analysis.

**TABLE 4:** Rates of abnormal cord insertion for twin–twin transfusion (TTTS) twins undergoing laser ablation vs non‐TTTS MC twins and rates of donor vs recipient TTTS twins undergoing laser.

Paper	Events out of total TTTS MC laser participants (*n*/*N*)	Events out of total non‐TTTS MC participants (*n*/*N*)	OR effect estimate (95% CI)	*I* ^2^
Rate of combined abnormal cord insertion (velamentous and marginal)				
Costa‐Castro (2016)^19^	310/435	370/513	0.96 (0.72–1.27)	
De Paepe (2005)^35^	11/30	53/148	1.04 (0.46–2.34)	
Lopriore (2007)^43^	69/152	51/126	1.22 (0.76–1.97)	
Yonetani (2015)^20^	24/60	242/654	1.13 (0.66–1.95)	
Total	677	1441	1.04 (0.66–1.29)	0%
Rate of velamentous cord insertion				
Costa‐Castro (2016)^19^	174/435	190/513	1.13 (0.87–1.47)	
Favre (2013)^38^	12/20	13/24	1.27 (0.38–4.22)	
Lopriore (2007)^43^	20/152	18/126	0.91 (0.46–1.80)	
Yonetani (2015)^20^	8/60	80/654	1.10 (0.51–2.41)	
Total	667	1317	1.11 (0.88–1.39)	0%
Rate of marginal cord insertion				
Costa‐Castro (2016)^19^	163/435	211/513	0.86 (0.66–1.11)	
Lopriore (2007)^43^	49/152	33/126	1.34 (0.79–2.26)	
Yonetani (2015)^20^	16/60	162/654	1.10 (0.61–2.01)	
Total	647	1293	0.96 (0.77–1.19)	20%

Paper	Events out of total donor twin participants (*n*/*N*)	Events out of total recipient twin participants (*n*/*N*)	OR effect estimate (95% CI)	*I* ^2^
Rate of combined abnormal cord insertion (velamentous and marginal)				
Lopriore (2007)^43^	48/76	21/76	4.49 (2.26–8.91)	
Saito (2019)^46^	2/6	0/6	7.22 (0.28–189.19)	
Starnes (2019)^48^	19/33	11/33	2.71 (1.00–7.38)	
Van Winden (2016)^49^	46/62	17/61	7.44 (3.35–16.53)	
Total	177	176	4.82 (3.06–7.58)	0%
Rate of velamentous cord insertion				
Chmait (2010)^34^	83/104	36/104	7.47 (3.99–13.97)	
Lopriore (2007)^43^	18/76	2/76	11.48 (2.56–51.50)	
Saito (2019)^46^	2/6	0/6	7.22 (0.28–189.19)	
Starnes (2019)^48^	12/33	8/33	1.79 (0.61–5.19)	
Van Winden (2016)^49^	23/62	6/61	5.41 (2.01–14.52)	
Total	281	280	5.76 (3.71–8.95)	35%
Rate of marginal cord insertion				
Lopriore (2007)^43^	30/76	19/76	1.96 (0.98–3.91)	
Starnes (2019)^48^	7/33	3/33	2.69 (0.63–11.49)	
Van Winden (2016)^49^	2362	11/61	2.68 (1.17–6.16)	
Total	171	170	2.28 (1.49–3.76)	0%

*Note*: Only the first author is given for each study. Filled color means data entry is not applicable.

Abbreviations: CI, confidence interval; MC, monochorionic; OR, odds ratio; TTTS, twin–twin transfusion syndrome.

Four studies compared the rates of combined abnormal cord insertion (velamentous and marginal) within MC twins with TTTS undergoing laser ablation vs MC twins uncomplicated by TTTS. Results were non‐significant (OR, 1.04 (95% CI, 0.84–1.29); *I* ^2=^0%). Studies comparing only velamentous or marginal cord insertion between these groups, results were additionally non‐significant (OR, 1.11 (95% CI, 0.88–1.39); *I* ^2=^0%) (four studies) and (OR, 0.96 (95% CI, 0.77–1.19); *I* ^2=^20%) (three studies), respectively. Results for each individual study can be visualized in Table 4.

Rates of IUFD and NND with respect to abnormal cord insertion could not be meta‐analyzed due to insufficient amounts of data within the included studies. Additionally, there were insufficient data within the included studies to compare abnormal cord insertion directly against the proportion of residual vascular anastomoses following laser ablation.

#### 3.2.3 Subgroup analysis

Rates of abnormal cord insertion within donor and recipient TTTS MC twins undergoing laser were discussed by several authors. Rates of combined, velamentous, and marginal cord insertion were all significantly greater for the donor twin vs the recipient twin for those complicated by TTTS which underwent laser ablation (OR, 4.82 (95% CI, 3.06–7.58); *I* ^2^ = 0%, four studies), (OR, 5.76 (95% CI, 3.71–8.95); *I* ^2^ = 35%, five studies), and (OR, 2.28 (95% CI, 1.39–3.76); *I* ^2^ = 0%, three studies) respectively. Individual study results can be visualized in Table 4.

**FIGURE 6:**
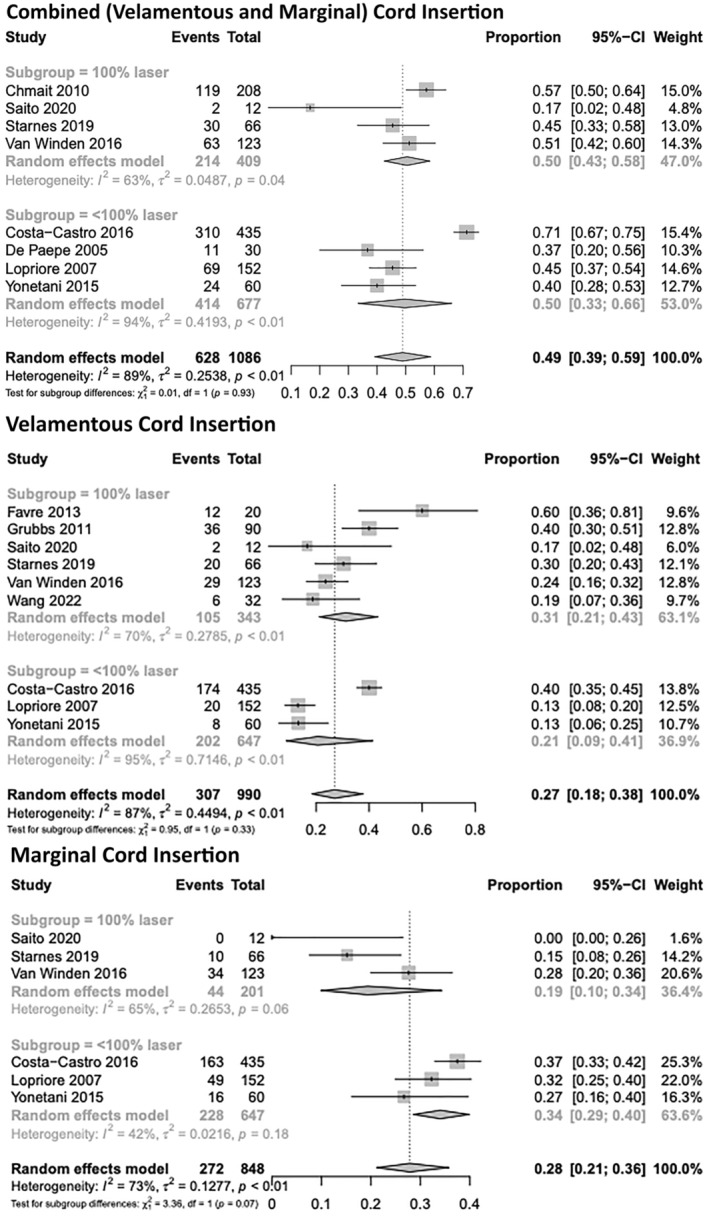
Individual study proportion rates for abnormal cord insertion (combined, velamentous, and marginal) within studies containing all participants undergoing laser ablation and studies containing fewer than 100% participants undergoing laser ablation.

Four studies examining the rates of abnormal cord insertion were noted to additionally include twins undergoing alternative interventions (amnio drainage and immediate induction of labor) or without intervention. For these studies, the percentages of TTTS MC twins undergoing laser ablation within the total cohort were 85%, 67%, 60%, and 80%, respectively.^19^, ^20^, ^35^, ^43^ Following removal of these studies proportional rates of combined, velamentous, and marginal cord insertion were 50% ((95% CI, 0.43–0.58); *I* ^2^ = 63%, four studies), 31% ((95% CI, 0.21–0.43); *I* ^2^ = 70%, six studies), and 19% ((95% CI, 0.10–0.34); *I* ^2^ = 65%, three studies), respectively. Subgroup proportional analysis of abnormal cord insertion for studies with the entire cohort undergoing laser vs studies containing <100% of the cohort undergoing laser ablation was not statistically different for combined (*p* = 0.93), velamentous (*p* = 0.33), or marginal (*p* = 0.07), respectively (Figure 6).

Removal of studies with less than 100% of the cohort undergoing laser continued to demonstrate significantly more combined and velamentous cord insertions within the donor twin compared to the recipient twin (OR, 5.09 (95% CI, 2.78–9.32); *I* ^2=^18%, three studies) and (OR, 5.23 [95% CI 3.29–8.31]; *I* ^2=^42%, four studies), respectively. Marginal cord insertion within the donor vs recipient twin could not be meta‐analyzed. Meta‐analysis of MC twins complicated by TTTS undergoing laser ablation vs MC twins uncomplicated by TTTS could not be performed across all cord insertion groups following the removal of studies with <100% of the cohort undergoing laser ablation.

#### 3.2.4 Risk of bias

The full risk of bias assessments for all included studies are detailed in Table S2, within Supporting Information. Twelve studies were graded as low risk and one a moderate risk of bias. The included RCT was low risk across all domains of the Cochrane RoB2 tool. The most common reasons for a reduction in score were not identifying confounding factors and loss of follow‐up not correctly recorded.

### 3.3 Additional placental outcomes

In addition to the above outcomes that have been discussed, four included studies additionally detailed post‐natal placental sharing and placental weight data.^4^, ^38^, ^41^, ^49^ Khalek et al.^41^ and Van Winden et al.^49^ commented on the mean placental donor shares of 45.3% (10–90) and 40.0% (20–70), respectively. Van Winden et al.^49^ noted that 21.9% of TTTS MC laser twins had a donor placental share <30%, whereas Favre et al.^38^ noted 35% of donors had a placental share <20%. Donor and recipient placental weights were described by two studies, with similar values. Donor placental weights were 291 g ± 124 and 311 g ± 154, respectively, while recipient placental weights were 396 g ± 133 and 408 g ± 148, respectively.^38^, ^49^ Van Winden et al.^49^ additionally compared fetal–placental weight ratio between TTTS MC twins and TTTS MC twins complicated with sIUGR and found that sIUGR was not an associating factor (*p* = 0.83). Wang et al.^4^ additionally noted that TTTS undergoing laser is not an influential factor on placental territory discordance ratio when compared to non‐TTTS MC twins (*p* = 0.236).

## 4 DISCUSSION

This review provides quantitative findings on post‐laser placental characteristics for TTTS MC twins. We demonstrate that nearly a quarter of all lasered placentas contained residual anastomoses. Residual anastomoses are significantly associated with the development of post‐laser TAPS, recurrent TTTS, IUFD, and NND. Placental positioning and Quintero staging are non‐significant.

Abnormal cord insertion was detected in approximately half of all TTTS MC twins that underwent laser. Abnormal cord insertion is more frequently seen within donor twins. Abnormal cord insertion is not significantly associated with the development of TTTS requiring laser.

Our review represents the first study to amalgamate data on residual anastomoses post‐laser for MC twins complicated by TTTS, including associated fetal outcomes. Residual anastomoses have been widely categorized into three main types: AA, AV, and VV. AA and VV anastomoses are more commonly superficial and classically bidirectional, yielding a protective effect for potential MC complications.^16^, ^57^ AV anastomoses are obligate unidirectional and typically more deeply located within the placental tissue.^57^ Consequently, adequate visualization during fetoscopy and complete ablation can be troublesome. As a result, residual anastomoses post‐laser can occur, which when uncompensated by the bilateral protective nature of AA/VV anastomoses, can increase the risk of developing post‐laser complications and adverse perinatal outcomes.^16^, ^42^, ^58^, ^59^

In our review, approximately 24% of placentas contained residual anastomoses, of which 75% contained AV anastomoses. Such a large proportion of residual unidirectional anastomoses, in collaboration with results demonstrating a significant increase in post‐laser MC complications, reveals that our findings align closely with prior pathophysiological explanations within the literature.^3^, ^16^, ^57^, ^58^ Importantly, this review demonstrates original insight by quantifying the relationship between residual anastomoses and post‐laser MC complications. This review enables clinicians the ability to clearly inform patients of potential post‐laser risks during pre‐ and post‐operative counseling, such that if a surgeon anticipates residual anastomoses are likely present post‐laser, then quantifiable risks presented within this review can help facilitate discussions regarding possible fetal outcomes. This review presents a strong association with recurrent TTTS and post‐laser TAPS when residual anastomoses following laser ablation are present. Clinicians may consider increased awareness and surveillance for these potential fetal complications during the post‐operative period to ensure earlier detection and subsequent antenatal management.

In recent years, the surgical technique for ablating anastomoses has adapted from the selective to Solomon method, aiming to coagulate the entire vascular equator and reduce numbers of residual anastomoses.^9^, ^60^ Prior literature has demonstrated a significant reduction in certain post‐laser MC complications and adverse perinatal outcomes with the Solomon technique.^7^, ^61^, ^62^ Within this review, many studies did not allude to the surgical technique used for ablation. However, it should be noted that many of the included studies would have predated the now recognized Solomon technique. While it is assumed that the Solomon technique reduces residual anastomoses, prior reviews, including ours, have been unable to determine the proportion of post‐laser residual anastomoses with each technique based on the status of existing literature. Importantly, the Solomon technique risks additionally ablating the bidirectional, protective AA/VV anastomoses. This may influence the propensity for adverse fetal outcomes to occur, particularly if AV residual anastomoses are present. Therefore, it is necessitated that future studies examining residual anastomoses clearly delineate the surgical technique used to enable future comparison and meta‐analysis. Additionally, future research endeavors should consider techniques to improve the selection of AV anastomosis intraoperatively.

Prior literature has frequently demonstrated a non‐significant association between TTTS and abnormal cord insertion.^19^, ^20^, ^21^, ^43^ These findings are consistent with our presented results. However, our findings provide a unique exploration into the relationship between twins specifically requiring laser and abnormal cord insertion. Interestingly, the most recent Royal College of Obstetricians and Gynecologists guideline for MC twins notes abnormal cord insertion is often associated with TTTS.^63^ However, studies cited within this guideline do not report a correlation.^64^, ^65^, ^66^, ^67^

While abnormal cord insertion may not be a critical determinant for the evolution of TTTS, including twins requiring laser treatment, we are unable to determine their association with residual anastomoses. Evidence has demonstrated that discordant cord insertions can distort the vascular equator along the chorionic surface.^68^, ^69^, ^70^ This can ultimately lead to difficulty in achieving complete intraoperative ablation of the anastomoses. Determining the association between abnormal cord insertion and residual anastomoses may allow clinicians to risk stratify women pre‐operatively and employ patient‐specific counseling advice for continued post‐operative management. A large prospective multicenter study is required to address this question. Additionally, Bonanni et al have recently demonstrated a significant association with abnormal cord insertion and adverse fetal outcomes. This highlights the importance of improving the detection of these abnormal placental anatomical features pre‐operatively, enabling timely intervention and improved operative planning.^70^

While it is important to demonstrate evidence regarding post‐laser TTTS placental architecture, it is equally important to consider alternative strategies to detect these placental characteristics pre‐operatively. An emerging interest within fetal medicine is the use of pre‐operative MRI.^71^ Torrents‐Barrena et al.^72^ proposed the first TTTS simulator for clinical use, allowing clinicians to pre‐operatively visualize umbilical cord insertions and placental anastomoses, allowing for detailed TTTS surgical planning. Lewi et al.^73^ and Luks et al.^74^ additionally evaluated the use of pre‐operative MRI, demonstrating an 89% accuracy for predicting twin placental volume distribution and enabling virtual simulation of the intertwin membrane location and placenta, respectively. Consequently, a detailed pre‐operative analysis of the intrauterine environment ensures clinicians are fully equipped for surgery, ensuring correct location of the umbilical cords and vascular equator, and maximizing complete intertwin anastomotic ablation.

To the best of our knowledge, this is the first systematic review that has explored outcomes of residual anastomoses and abnormal cord insertion within post‐laser TTTS twins. An initial scoping search, a thorough formal literature search, and a robust methodological strategy, including a deep assessment of outcomes, are the main strengths of this review. Studies were additionally validated by two authors including consultation with a third author if any conflicts occurred. This intended to improve the accuracy of included studies and the objectivity of the findings. Many included studies were retrospective, non‐randomized, containing small sample sizes while demonstrating a wide heterogeneity in methodological quality regarding placental analysis and participant inclusion criteria. These represent the main weaknesses of this review.

Many studies assessing residual anastomoses only included dual survivors, potentially increasing the risk of selection bias. In turn, this may have underestimated the risks associated with residual anastomoses. Additionally, many studies automatically excluded single‐ or dual‐fetal demise due to the potential risk of placental maceration. This may introduce further selection and publication bias, given that those fetuses that were demised are likely to have had residual anastomoses. However, our subgroup analyses comparing survivors vs fetal demise aimed to rectify this concern.

Given the nature of certain outcomes presented within small numbers within individual studies, there was potential for publication bias. Consequently, this limits the reliability of certain forest plots examining adverse fetal outcomes (left side limited to 0 value for outcome rates and wide confidence intervals). Furthermore, many included studies originated from high‐income countries, where laser ablation is routinely available within tertiary centers, with rigorous TTTS pathways, including an established pathology department for placental analysis. Surgical success and subsequent fetal outcomes may, however, differ for middle‐/lower‐income countries whereby these services may not be as established due to lack of healthcare resources. This potentially introduces publication bias within this review. Consequently, the results presented here may not achieve full worldwide generalizability. Low‐/middle‐income countries may need to conduct their own local prospective studies to facilitate patients with region‐specific outcome information.

In addition, included studies demonstrated a variation in their reporting techniques for each group examined, whether that be presenting the raw data, the mean and SD, or the median and range. Consequently, the values we present for the overall anastomoses mean, including vascular subtypes and diameters, may slightly differ from the true values if all raw data were provided for calculation. However, despite these restrictions, this paper represents the most comprehensive and up‐to‐date review of placental characteristics for post‐laser TTTS MC twins.

## 5 CONCLUSION

Residual anastomoses were detected within approximately a quarter of all post‐laser TTTS twins. Residual anastomoses are significantly associated with an increased rate of recurrent TTTS, post‐laser TAPS, IUFD, and NND. Abnormal cord insertion was detected in almost half of all post‐laser TTTS twins. Abnormal cord insertion is not an influencing factor for TTTS development within twins requiring laser ablation. A large prospective study is necessitated to assess the relationship between abnormal cord insertion and residual anastomoses post‐laser.

## AUTHOR CONTRIBUTIONS

Jack Hamer conceived the idea for the review, performed the literature search, screened texts, performed the risk of bias assessment, performed the statistical analysis, and wrote the draft and final manuscript. Nashwa Eltaweel conceived the idea for the review, screened texts as second reviewer, performed the risk of bias assessment, reviewed/edited the draft manuscript, and approved/appraised the final submission. Rebecca Man and Matilde Rogerson performed the statistical analysis for the review, reviewed/edited the draft manuscript, and approved/appraised the final submission. Victoria Hodgetts Morton, Katie Morris, and Tamas Marton reviewed and edited the draft manuscripts and approved/appraised the final submission. Leo Gurney conceived the idea for the review, acted third reviewer to resolve literature inclusion conflicts, and reviewed/edited the final manuscript.

## CONFLICT OF INTEREST STATEMENT

None.

## Supporting information


Table S1.

Table S2.

Table S3.

## References

[1] Benoit RM , Baschat AA . Twin‐to‐twin transfusion syndrome: prenatal diagnosis and treatment. Am J Perinatol. 2014;31:583‐594. 24858318 10.1055/s-0034-1372428

[2] Bamberg C , Hecher K . Twin‐to‐twin transfusion syndrome: controversies in the diagnosis and management. Best Pract Res Clin Obstet Gynaecol. 2022;84:143‐154. 35589537 10.1016/j.bpobgyn.2022.03.013

[3] Shanahan MA , Bebbington MW . Placental anatomy and function in twin gestations. Obstet Gynecol Clin N Am. 2020;47:99‐116. 10.1016/j.ogc.2019.10.010 32008674

[4] Wang X , Li L , Yuan P , Zhao Y , Wei Y . Effect of fetoscopic laser surgery on the placental characteristics and birth‐weight discordance of twins with twin‐to‐twin transfusion syndrome. Front Med. 2022;9:942816. 10.3389/fmed.2022.942816 PMC9556886 36250079

[5] Quintero RA , Morales WJ , Allen MH , Bornick PW , Johnson PK , Kruger M . Staging of twin‐twin transfusion syndrome. J Perinatol. 1999;19:550‐555. 10645517 10.1038/sj.jp.7200292

[6] Di Mascio D , Khalil A , D'Amico A , et al. Outcome of twin–twin transfusion syndrome according to Quintero stage of disease: systematic review and meta‐analysis. Ultrasound Obstet Gynecol. 2020;56:811‐820. 32330342 10.1002/uog.22054

[7] D'Antonio F , Herrera M , Oronzii L , Khalil A . Solomon technique *vs* selective fetoscopic laser photocoagulation for twin–twin transfusion syndrome: systematic review and meta‐analysis of maternal and perinatal outcomes. Ultrasound Obstet Gynecol. 2022;60:731‐738. 36240516 10.1002/uog.26095

[8] Senat MV , Deprest J , Boulvain M , Paupe A , Winer N , Ville Y . Endoscopic laser surgery versus serial amnioreduction for severe twin‐to‐twin transfusion syndrome. N Engl J Med. 2004;351:136‐144. 15238624 10.1056/NEJMoa032597

[9] Slaghekke F , Lopriore E , Lewi L , et al. Fetoscopic laser coagulation of the vascular equator versus selective coagulation for twin‐to‐twin transfusion syndrome: an open‐label randomised controlled trial. Lancet. 2014;383:2144‐2151. 24613024 10.1016/S0140-6736(13)62419-8

[10] Feng S , Li G , Yin P , Zhu T , Cheng C , Dong L . Relationship between the types and diameters of residual vessels and secondary TAPS after Fetoscopic laser surgery for TTTS. Z Geburtshilfe Neonatol. 2022;226:240‐244. 35998656 10.1055/a-1862-8571

[11] Knijnenburg PJC , Slaghekke F , Tollenaar LSA , et al. Incidence of and risk factors for residual anastomoses in twin‐twin transfusion syndrome treated with laser surgery: a 15‐year single‐center experience. Fetal Diagn Ther. 2019;45:13‐20. 29332067 10.1159/000485932

[12] Konno H , Murakoshi T , Matsushita M . The roles of superficial anastomoses in twin‐twin transfusion syndrome. Placenta. 2019;82:5‐9. 31174627 10.1016/j.placenta.2019.05.003

[13] Quintero RA , Chmait RH , Bornick PW , Kontopoulos EV . Trocar‐assisted selective laser photocoagulation of communicating vessels: a technique for the laser treatment of patients with twin‐twin transfusion syndrome with inaccessible anterior placentas. J Matern Fetal Neonatal Med. 2010;23:330‐334. 19941443 10.3109/14767050903177177

[14] Lopriore E , Middeldorp JM , Oepkes D , Klumper FJ , Walther FJ , Vandenbussche FP . Residual anastomoses after fetoscopic laser surgery in twin‐to‐twin transfusion syndrome: frequency, associated risks and outcome. Placenta. 2007;28:204‐208. 16644009 10.1016/j.placenta.2006.03.005

[15] van den Wijngaard JP , Lopriore E , van der Salm SM , et al. Deep‐hidden anastomoses in monochorionic twin placentae are harmless. Prenat Diagn. 2007;27:233‐239. 17186565 10.1002/pd.1652

[16] Hubinont C , Lewi L , Bernard P , Marbaix E , Debiève F , Jauniaux E . Anomalies of the placenta and umbilical cord in twin gestations. Am J Obstet Gynecol. 2015;213:S91‐S102. 26428508 10.1016/j.ajog.2015.06.054

[17] Sato Y , Benirschke K . Increased prevalence of fetal thrombi in monochorionic‐twin placentas. Pediatrics. 2006;117:e113‐e117. 16361224 10.1542/peds.2005-1501

[18] Ebbing C , Kiserud T , Johnsen SL , Albrechtsen S , Rasmussen S . Prevalence, risk factors and outcomes of velamentous and marginal cord insertions: a population‐based study of 634,741 pregnancies. PLoS One. 2013;8:e70380. 23936197 10.1371/journal.pone.0070380 PMC3728211

[19] Costa‐Castro T , Zhao DP , Lipa M , et al. Velamentous cord insertion in dichorionic and monochorionic twin pregnancies—does it make a difference? Placenta. 2016;42:87‐92. 27238718 10.1016/j.placenta.2016.04.007

[20] Yonetani N , Ishii K , Kawamura H , Mabuchi A , Hayashi S , Mitsuda N . Significance of Velamentous cord insertion for twin‐twin transfusion syndrome. Fetal Diagn Ther. 2015;38:276‐281. 25925425 10.1159/000381639

[21] Kalafat E , Thilaganathan B , Papageorghiou A , Bhide A , Khalil A . Significance of placental cord insertion site in twin pregnancy. Ultrasound Obstet Gynecol. 2018;52:378‐384. 28976606 10.1002/uog.18914

[22] De Paepe ME , Shapiro S , Young L , Luks FI . Placental characteristics of selective birth weight discordance in diamniotic‐monochorionic twin gestations. Placenta. 2010;31:380‐386. 20303588 10.1016/j.placenta.2010.02.018

[23] Moher D , Liberati A , Tetzlaff J , Altman DG , PRISMA Group . Preferred reporting items for systematic reviews and meta‐analyses: the PRISMA statement. PLoS Med. 2009;6:e1000097. 19621072 10.1371/journal.pmed.1000097 PMC2707599

[24] Deeks JJ , Higgins JPT , Altman DG . Chapter 10: analysing data and undertaking meta‐analyses. In: Higgins JPT , Thomas J , Chandler J , et al., eds. Cochrane Handbook for Systematic Reviews of Interventions version 6.4 (updated August 2023). Cochrane; 2023.

[25] Review Manager (RevMan) Version 5.4. T.C. Collaboration, Editor. 2020. Accessed January 2, 2024. https://www.revman.cochrane.org

[26] Higgins JP , Thompson SG , Deeks JJ , Altman DG . Measuring inconsistency in meta‐analyses. BMJ. 2003;327:557‐560. 12958120 10.1136/bmj.327.7414.557 PMC192859

[27] Hozo SP , Djulbegovic B , Hozo I . Estimating the mean and variance form the median, range and the size of a sample. BMC Med Res Methodol. 2005;5:1‐10. 15840177 10.1186/1471-2288-5-13 PMC1097734

[28] Wan X , Wang W , Liu J , Tong T . Estimating the sample mean and standard deviation from the sample size, median, range and/or interquartile range. BMC Med Res Methodol. 2014;14:135. 25524443 10.1186/1471-2288-14-135 PMC4383202

[29] Higgins JPT , Li T , Deeks JJ . Chapter 6: choosing effect measures and computing estimates of effect. In: Higgins JPT , Thomas J , Chandler J , et al., eds. Cochrane Handbook for Systematic Reviews of Interventions version 6.4 (updated August 2023). Cochrane; 2023.

[30] Team RC . R: A Language and Environment for Statistical Computing. R Foundation for Statistical Computing; 2013 https://www.R‐project.org/

[31] JBI . Critical appraisal tools. 2023. Accessed February 12, 2024. https://jbi.global/critical‐appraisal‐tools

[32] Sterne JAC , Savović J , Page MJ , et al. RoB 2: a revised tool for assessing risk of bias in randomised trials. BMJ. 2019;366:l4898. 31462531 10.1136/bmj.l4898

[33] Bermúdez C , Becerra CH , Bornick PW , Allen MH , Arroyo J , Quintero RA . Placental types and twin‐twin transfusion syndrome. Am J Obstet Gynecol. 2002;187:489‐494. 12193948 10.1067/mob.2002.124280

[34] Chmait RH , Assaf SA , Benirschke K . Residual vascular communications in twin‐twin transfusion syndrome treated with sequential laser surgery: frequency and clinical implications. Placenta. 2010;31:611‐614. 20451248 10.1016/j.placenta.2010.04.006

[35] De Paepe ME , DeKoninck P , Friedman RM . Vascular distribution patterns in monochorionic twin placentas. Placenta. 2005;26:471‐475. 15950060 10.1016/j.placenta.2004.06.014

[36] De Paepe ME , Friedman RM , Poch M , Hansen K , Carr SR , Luks FI . Placental findings after laser ablation of communicating vessels in twin‐to‐twin transfusion syndrome. Pediatr Dev Pathol. 2004;7:159‐165. 15022066 10.1007/s10024-003-9099-3

[37] Egawa M , Hayashi S , Matsuoka K , et al. Residual vascular communications after fetoscopic laser surgery in twin‐twin transfusion syndrome. Frequency and outcome. Placenta. 2012;33:A104.

[38] Favre R , Koch A , Weingertner AS , et al. Vascular pattern in monochorionic placentas with spontaneous TAPS and TTTS with residual anastomoses after laser: a case‐control study. Prenat Diagn. 2013;33:979‐982. 23744723 10.1002/pd.4177

[39] Gratacós E , Lewi L , Carreras E , et al. Incidence and characteristics of umbilical artery intermittent absent and/or reversed end‐diastolic flow in complicated and uncomplicated monochorionic twin pregnancies. Ultrasound Obstet Gynecol. 2004;23:456‐460. 15133795 10.1002/uog.1013

[40] Grubbs BH , Benirschke K , Korst LM , Llanes A , Yedigarova L , Chmait RH . Role of low placental share in twin‐twin transfusion syndrome complicated by intrauterine growth restriction. Placenta. 2011;32:616‐618. 21664690 10.1016/j.placenta.2011.05.009

[41] Khalek N , Villa A , Soni S , et al. Qualitative and quantitative review of placental pathology in pregnancies affected by twin twin transfusion syndrome undergoing selective laser photocoagulation. Am J Obstet Gynecol. 2014;210:S86.

[42] Lewi L , Jani J , Cannie M , et al. Intertwin anastomoses in monochorionic placentas after fetoscopic laser coagulation for twin‐to‐twin transfusion syndrome: is there more than meets the eye? Am J Obstet Gynecol. 2006;194:790‐795. 16522414 10.1016/j.ajog.2005.08.062

[43] Lopriore E , Sueters M , Middeldorp JM , Oepkes D , Walther FJ , Vandenbussche FP . Velamentous cord insertion and unequal placental territories in monochorionic twins with and without twin‐to‐twin‐transfusion syndrome. Am J Obstet Gynecol. 2007;196:159‐159.e5. 10.1016/j.ajog.2006.10.865 17306663

[44] Lopriore E , Slaghekke F , Middeldorp JM , Klumper FJ , Oepkes D , Vandenbussche FP . Residual anastomoses in twin‐to‐twin transfusion syndrome treated with selective fetoscopic laser surgery: localization, size, and consequences. Am J Obstet Gynecol. 2009;201:66‐66.e4. 10.1016/j.ajog.2009.01.010 19306965

[45] Peeters S , Slaghekke F , Middeldorp J , Klumper F , Lopriore E , Oepkes D . Residual anastomoses after fetoscopic laser coagulation in twin‐to‐twin transfusion syndrome: a learning curve. Am J Obstet Gynecol. 2012;206:S285.

[46] Saito M , Tokunaka M , Takita H , et al. Impact of first trimester determination of abnormal cord insertion on twin‐to‐twin transfusion syndrome and other adverse outcomes in monochorionic diamniotic twins: a retrospective cohort study. Prenat Diagn. 2020;40:507‐513. 31875322 10.1002/pd.5633

[47] Slaghekke F , Lewi L , Middeldorp JM , et al. Residual anastomoses in twin‐twin transfusion syndrome after laser: the Solomon randomized trial. Am J Obstet Gynecol. 2014;211:285. 10.1016/j.ajog.2014.05.012 24813598

[48] Starnes SE , Nardi F , Fitchev P , et al. Influence of maternal obesity and metabolic and vascular mediators in twin‐twin transfusion syndrome. Reprod Biol. 2019;19:165‐172. 31147266 10.1016/j.repbio.2019.05.002

[49] Van Winden KR , Quintero RA , Kontopoulos EV , Korst LM , Llanes A , Chmait RH . Decreased total placental mass found in twin‐twin transfusion syndrome gestations with selective growth restriction. Fetal Diagn Ther. 2016;40:116‐122. 26784929 10.1159/000442153

[50] Zhao DP , Peeters SH , Middeldorp JM , Klumper FJ , Oepkes D , Lopriore E . Laser surgery in twin‐twin transfusion syndrome with proximate cord insertions. Placenta. 2013;34:1159‐1162. 24157353 10.1016/j.placenta.2013.10.004

[51] Hecher K , Bambery C . Complicated monochorionic twin pregnancies. Gynakol Prax. 2020;46:559‐568.

[52] Shao H , Wei Y , Yuan P , Guo X , Wang Y , Zhao Y . Research of placental vascular distribution and clinical outcome in monochorionic twins. Zhonghua Fu Chan Ke Za Zhi. 2013;48:411‐415. 24103118

[53] Sun L , Zou G , Yan Y , et al. Pregnancy outcome after fetoscopic laser photocoagulation for twin‐twin transfusion syndrome: experience of an emerging center from China. Zhonghua Fu Chan Ke Za Zhi. 2014;49:404‐409. 25169629

[54] Wang XJ , Li LY , Yuan PB , Wang Y , Zhao YY , Wei Y . Effect of placental vascular distribution on residual anastomoses after fetoscopic laser surgery for twin to twin transfusion syndrome. Zhonghua Fu Chan Ke Za Zhi. 2021;56:171‐177. 33874711 10.3760/cma.j.cn112141-20200903-00689

[55] Wang X , Wei Y , Yuan P , Zhao Y . Placental characteristics of twin‐to‐twin transfusion syndrome. Zhonghua Yi Xue Za Zhi. 2015;95:1323‐1327. 26081663

[56] Yang L , Shao H , Yuan P , Guo X , Zhang X , Zhao Y . Expressions of HIF‐alpha and its target gene in monochorionic twin placentas with twin‐twin transfusion syndrome. Beijing Da Xue Xue Bao. 2011;43:792‐797. 22178822

[57] Couck I , Lewi L . The placenta in twin‐to‐twin transfusion syndrome and twin anemia polycythemia sequence. Twin Res Hum Genet. 2016;19:184‐190. 27098457 10.1017/thg.2016.29

[58] Lewi L , Deprest J , Hecher K . The vascular anastomoses in monochorionic twin pregnancies and their clinical consequences. Am J Obstet Gynecol. 2013;208:19‐30. 23103301 10.1016/j.ajog.2012.09.025

[59] Denbow ML , Cox P , Taylor M , Hammal DM , Fisk NM . Placental angioarchitecture in monochorionic twin pregnancies: relationship to fetal growth, fetofetal transfusion syndrome, and pregnancy outcome. Am J Obstet Gynecol. 2000;182:417‐426. 10694346 10.1016/s0002-9378(00)70233-x

[60] Slaghekke F , Oepkes D . Solomon technique versus selective coagulation for twin‐twin transfusion syndrome. Twin Res Hum Genet. 2016;19:217‐221. 27203607 10.1017/thg.2016.25

[61] Shamshirsaz AA , Chmait RH , Stirnemann J , et al. Solomon versus selective fetoscopic laser photocoagulation for twin‐twin transfusion syndrome: a systematic review and meta‐analysis. Prenat Diagn. 2023;43:72‐83. 36184777 10.1002/pd.6246

[62] Dhillon RK , Hillman SC , Pounds R , Morris RK , Kilby MD . Comparison of Solomon technique with selective laser ablation for twin‐twin transfusion syndrome: a systematic review. Ultrasound Obstet Gynecol. 2015;46:526‐533. 25677883 10.1002/uog.14813

[63] Kilby MD , Bricker L on behalf of the Royal College of Obstetricians and Gynaecologists . Management of monochorionic twin pregnancy. BJOG. 2016;124:e1‐e45.27862859

[64] Umur A , van Gemert MJ , Nikkels PG . Monoamniotic‐versus diamniotic‐monochorionic twin placentas: anastomoses and twin‐twin transfusion syndrome. Am J Obstet Gynecol. 2003;189:1325‐1329. 14634563 10.1067/s0002-9378(03)00811-1

[65] Zhao DP , de Villiers SF , Slaghekke F , et al. Prevalence, size, number and localization of vascular anastomoses in monochorionic placentas. Placenta. 2013;34:589‐593. 23639577 10.1016/j.placenta.2013.04.005

[66] Chang YL , Chang SD , Chao AS , Hsieh PC , Wang CN , Wang TH . Clinical outcome and placental territory ratio of monochorionic twin pregnancies and selective intrauterine growth restriction with different types of umbilical artery Doppler. Prenat Diagn. 2009;29:253‐256. 19248041 10.1002/pd.2193

[67] de Villiers SF , Slaghekke F , Middeldorp JM , Walther FJ , Oepkes D , Lopriore E . Arterio‐arterial vascular anastomoses in monochorionic placentas with and without twin‐twin transfusion syndrome. Placenta. 2012;33:652‐654. 22652047 10.1016/j.placenta.2012.05.003

[68] Couck I , Jespers A , Deprest J , Lewi L . The vascular equator in monochorionic twin placentas. Placenta. 2020;99:193‐196. 32988575 10.1016/j.placenta.2020.05.008

[69] Chu S , Mao Q , Shapiro S , Luks SL , De Paepe ME . Correlation between cord insertion type and chorionic villus vascularization of the co‐twin in diamniotic‐monochorionic twin pregnancies. Early Hum Dev. 2013;89:243‐247. 23419860 10.1016/j.earlhumdev.2013.01.009

[70] Bonanni G , Airoldi C , Romanzi F , et al. The impact of placental anastomoses and umbilical cord insertions' sites on monochorionic twin pregnancy outcomes: evidence from color‐dye injection studies. Placenta. 2023;143:110‐116. 37879258 10.1016/j.placenta.2023.10.007

[71] van der Schot AM , Sikkel E , Spaanderman MEA , Vandenbussche FPHA . Computer‐assisted fetal laser surgery in the treatment of twin‐to‐twin transfusion syndrome: recent trends and prospects. Prenat Diagn. 2022;42:1225‐1234. 35983630 10.1002/pd.6225 PMC9541851

[72] Torrents‐Barrena J , López‐Velazco R , Piella G , et al. TTTS‐GPS: patient‐specific preoperative planning and simulation platform for twin‐to‐twin transfusion syndrome fetal surgery. Comput Methods Prog Biomed. 2019;179:104993. 10.1016/j.cmpb.2019.104993 31443866

[73] Lewi L , Cannie M , Vandecaveye V , et al. OC22.08: feasibility of magnetic resonance imaging as a preoperative guidance tool for laser coagulation in twin‐twin transfusion syndrome. Ultrasound Obstet Gynecol. 2005;26:346.

[74] Luks FI , Carr SR , Ponte B , Rogg JM , Tracy TF Jr . Preoperative planning with magnetic resonance imaging and computerized volume rendering in twin‐to‐twin transfusion syndrome. Am J Obstet Gynecol. 2001;185:216‐219. 11483931 10.1067/mob.2001.115111

