# Supplementary material for: Placental architectural characteristics following laser ablation within monochorionic twins complicated by twin–twin transfusion syndrome: A systematic review and meta‐analysis of outcomes
Source: Acta Obstet Gynecol Scand. 2024 Jun 14;103(11):2130–46. doi: 10.1111/aogs.14891 (PMC11502458; doi:10.1111/aogs.14891)
Supplement: Supplementary file 1 — Table S1. [file AOGS-103-2130-s001.docx]

Table S1: Search Strategy.

Database: MEDLINE® and Embase®

August 2023

| Set# | Search Term | Results |
| --- | --- | --- |
| S1 | monochorionic.mp. [mp=ti, ab, hw, tn, ot, dm, mf, dv, kf, fx, dq, bt, nm, ox, px, rx, an, ui, sy, ux, mx] | 7831 |
| S2 | MC.mp. [mp=ti, ab, hw, tn, ot, dm, mf, dv, kf, fx, dq, bt, nm, ox, px, rx, an, ui, sy, ux, mx] | 89947 |
| S3 | monochorionicity.mp. [mp=ti, ab, hw, tn, ot, dm, mf, dv, kf, fx, dq, bt, nm, ox, px, rx, an, ui, sy, ux, mx] | 314 |
| S4 | s1 or s2 or s3 | 96973 |
| S5 | TTTS.mp. [mp=ti, ab, hw, tn, ot, dm, mf, dv, kf, fx, dq, bt, nm, ox, px, rx, an, ui, sy, ux, mx] | 2973 |
| S6 | twin to twin transfusion syndrome.mp. [mp=ti, ab, hw, tn, ot, dm, mf, dv, kf, fx, dq, bt, nm, ox, px, rx, an, ui, sy, ux, mx] | 5917 |
| S7 | twin-twin transfusion syndrome.mp. [mp=ti, ab, hw, tn, ot, dm, mf, dv, kf, fx, dq, bt, nm, ox, px, rx, an, ui, sy, ux, mx] | 4823 |
| S8 | FFTS.mp. [mp=ti, ab, hw, tn, ot, dm, mf, dv, kf, fx, dq, bt, nm, ox, px, rx, an, ui, sy, ux, mx] | 373 |
| S9 | feto-fetal transfusion syndrome.mp. [mp=ti, ab, hw, tn, ot, dm, mf, dv, kf, fx, dq, bt, nm, ox, px, rx, an, ui, sy, ux, mx] | 121 |
| S10 | s5 or s6 or s7 or s8 or s9 | 6638 |
| S11 | placenta.mp. [mp=ti, ab, hw, tn, ot, dm, mf, dv, kf, fx, dq, bt, nm, ox, px, rx, an, ui, sy, ux, mx] | 236430 |
| S12 | placental vasculature.mp. [mp=ti, ab, hw, tn, ot, dm, mf, dv, kf, fx, dq, bt, nm, ox, px, rx, an, ui, sy, ux, mx] | 977 |
| S13 | cord insertion.mp. [mp=ti, ab, hw, tn, ot, dm, mf, dv, kf, fx, dq, bt, nm, ox, px, rx, an, ui, sy, ux, mx] | 1614 |
| S14 | placental anastomosis.mp. [mp=ti, ab, hw, tn, ot, dm, mf, dv, kf, fx, dq, bt, nm, ox, px, rx, an, ui, sy, ux, mx] | 45 |
| S15 | s11 or s12 or s13 or s14 | 237030 |
| S16 | S4 and s10 and s15 | 1060 |
