# Supplementary material for: Placental architectural characteristics following laser ablation within monochorionic twins complicated by twin–twin transfusion syndrome: A systematic review and meta‐analysis of outcomes
Source: Acta Obstet Gynecol Scand. 2024 Jun 14;103(11):2130–46. doi: 10.1111/aogs.14891 (PMC11502458; doi:10.1111/aogs.14891)
Supplement: Supplementary file 2 — Table S2. [file AOGS-103-2130-s003.docx]

Table S2: Summary of characteristics for included studies.

| **Title/Year** | **Study Design** | **Country** | **Population Assessed** | **Exclusion** | **Intervention** | **Comparison** | **Outcomes** | **Risk of Bias** | **Summary** |
| --- | --- | --- | --- | --- | --- | --- | --- | --- | --- |
| Bermudez (2002)^33^ | Retrospective cohort | USA | 105 placentas from TTTS MC twins treated with selective laser technique. 26 placentas from non TTTS MC twins | 45 placentas not suitable for analysis due to tissue fragmentation | TTTS MC twins undergoing laser ablation | Non TTTS MC twins | Residual anastomoses present | 9/11 – low risk of bias | Strategies to deal with confounding variables and address incomplete follow-up not applicable |
| Chmait (2010)^34^ | Retrospective case series | USA | 105 placentas from TTTS MC twins treated with selective laser technique | Intrauterine fetal demise and placentas too disrupted for correct analysis | TTTS MC twins undergoing laser ablation | \ | Abnormal cord insertion (single number combing both marginal and velamentous) and residual anastomoses present | 10/10 – low risk of bias | \ |
| Costa-Castro (2016) ^19^ | Retrospective cohort | Netherlands | 552 placentas from dichorionic twins. 463 placentas from TTTS MC twins, of which 393 treated with laser (85%). 536 placentas from non TTTS MC twins | MC twins treated with selective feticide, TRAP, monoamniotic twins and higher multiples | TTTS MC twins undergoing laser ablation. 435 out of 463 could be assessed | Non TTTS MC twins. 513 out of 536 could be assessed | Abnormal cord insertion (velamentous and marginal) | 7/11 – moderate risk of bias | Confounding factors were not identified, reasons for loss of follow-up was not recorded |
| De Paepe (2005)^35^ | Retrospective cohort | USA | 15 placentas from TTTS MC twins. 9 out of 15 treated with laser (60%). 74 placentas from non TTTS MC twins | Not clearly noted | TTTS MC twins undergoing laser ablation | Non TTTS MC twins | Abnormal cord insertion (single number combing both marginal and velamentous) | 8/11 – low risk of bias | Confounding factors were not identified. Strategies to deal with confounding variables and address incomplete follow-up not applicable |
| De Paepe (2004)^36^ | Retrospective case series | USA | 10 placentas from TTTS MC twins treated with selective laser technique | \ | TTTS MC twins undergoing laser ablation. 2 out of the 10 could not be assessed | \ | Residual anastomoses present | 8/10 – low risk of bias | No clear exclusion criteria mentioned, and inclusion was not complete |
| Egawa (2012)^37^ | Retrospective case series | Japan | 115 placentas from TTTS MC twins treated with laser | Not clearly noted but implies damaged placentas from single intrauterine demise | TTTS MC twins undergoing laser ablation | \ | Residual anastomoses present | 8/10 – low risk of bias | No clear exclusion criteria mentioned and no clear reporting of participant demographics |
| Favre (2013)^38^ | Prospective cohort | France | 20 placentas from TTTS MC twins treated with laser. 12 placentas from TAPS twins. 24 placentas from non TTTS MC twins | Not clearly noted | TTTS MC twins undergoing laser ablation | Non TTTS MC twins | Abnormal cord insertion (velamentous) and residual anastomoses present | 9/11 – low risk of bias | Strategies to deal with confounding variables and address incomplete follow-up not applicable |
| Feng (2022)^10^ | Retrospective cohort | China | 31 placentas from TTTS MC twins treated with laser. 9 placentas from TTTS MC twins with secondary TAPS undergoing laser | Damaged or incomplete umbilical cords affecting injection, formalin-contaminated placentae or placenta with single fetal survival | TTTS MC twins undergoing laser ablation | TTTS MC twins with secondary TAPS undergoing laser | Residual anastomoses present | 7/11 – moderate risk of bias | Exposures measured were unclear for validity and reliability. Follow-up was unclear and strategies to address incomplete follow up was unclear |
| Gratacos (2004)^39^ | Prospective cohort | Spain | 50 placentas from TTTS MC twins treated with laser followed by amniodrainage. 40 placentas with sIUGR. 80 placentas from non TTTS MC twins | Not clearly noted | TTTS MC twins undergoing laser ablation | Non TTTS MC twins and twins with sIUGR | Residual anastomoses present – only AA. | 8/11 – low risk of bias | No clear exclusion criteria mentioned. Strategies to deal with confounding variables and address incomplete follow-up not applicable |
| Grubbs (2011)^40^ | Prospective case series | USA | 90 placentas from TTTS MC twins undergoing laser | Intrauterine demise (single or double) and monoamniotic twins | TTTS MC twins undergoing laser ablation | \ | Abnormal cord insertion (velamentous) | 8/11 – low risk of bias | Study did not have complete inclusion of participants or clear reporting of demographics |
| Khalek (2014)^41^ | Retrospective case series | USA | 112 placentas from TTTS MC twins undergoing the selective laser technique | Intrauterine demise (single or double) | TTTS MC twins undergoing laser ablation | \ | Residual anastomoses present | 4/10 – high risk of bias | Unclear reporting of inclusion criteria, unclear if condition was measured in a reliable manner, unclear methods for identifying the condition, no clear reporting of demographics |
| Knijnenburg (2019)^11^ | Retrospective case series | Netherlands | 371 placentas from TTTS MC twins undergoing laser. 213 placentas undergoing the Solomon technique, 158 placentas undergoing the selective technique | 209 single or double intrauterine demise. 5 placentas excluded for fixation of the placenta, 11 placentas excluded for damage, 85 placentas lost | TTTS MC twins undergoing laser ablation | \ | Residual anastomoses present | 10/10 – low risk of bias | \ |
| Konno (2019)^12^ | Retrospective cohort | Japan | 152 placentas from TTTS MC twins undergoing laser | 32 placentas excluded due to infarction after fetal demise or placental destruction | TTTS MC twins undergoing laser ablation with dual survival | TTTS MC twins undergoing laser ablation with single or double demise | Residual anastomoses present | 9/11 – low risk of bias | Strategies to address confounding factors and incomplete follow up were not applicable |
| Lewi (2006)^42^ | Retrospective cohort | \ | 50 placentas from TTTS MC twins undergoing the selective laser technique | 5 placentas excluded due to tissue fragmentation, 1 placenta excluded due to fixation in formalin | TTTS MC twins undergoing laser ablation with dual survival | TTTS MC twins undergoing laser ablation with double demise | Residual anastomoses present | 8/11 – low risk of bias | Strategies to address confounding factors was unclear. Strategies to address confounding factors and incomplete follow up were not applicable |
| Lopriore (2007)^43^ | Retrospective cohort | Netherlands | 76 placentas from TTTS MC twins. 61/76 treated with laser (80%). 63 placentas from non TTTS MC twins | 9 placentas excluded due to placental maceration, 3 placentas excluded due to placental fragmentation, 1 placenta excluded for fixation in formalin, 9 placentas lost or destroyed | TTTS MC twins undergoing laser ablation | Non TTTS MC twins | Abnormal cord insertion (velamentous and marginal) | 9/11 – low risk of bias | Strategies to address confounding factors and incomplete follow up were not applicable |
| Lopriore (2009)^44^ | Retrospective case series | Netherlands | 77 placentas from TTTS MC twins undergoing the selective laser technique | 41 placentas excluded due to intrauterine fetal demise, 14 placentas excluded due to entire coagulation of the equator, 6 placentas excluded due to damaged placentas, 4 placentas excluded due to incomplete surgery | TTTS MC twins undergoing laser ablation | \ | Residual anastomoses present | 10/10 – low risk of bias | \ |
| Peeters (2012)^45^ | Retrospective case series | Netherlands | 108 placentas from TTTS MC twins undergoing laser | Single or double intrauterine fetal demise | TTTS MC twins undergoing laser ablation | \ | Residual anastomoses present | 7/10 – moderate risk of bias | Unclear reporting of complete inclusion. No clear reporting of demographics or outcomes |
| Quintero (2010)^13^ | Retrospective case series | USA | 143 placentas from TTTS MC twins undergoing the selective laser technique | Triplet and monoamniotic pregnancies were excluded | TTTS MC twins undergoing laser ablation. | \ | Residual anastomoses present | 10/10 – low risk of bias | \ |
| Saito (2020)^46^ | Retrospective cohort | Japan | 6 placentas from TTTS MC twins undergoing laser and 5 placentas from TTTS MC twins not undergoing laser | Not clearly noted | TTTS MC twins undergoing laser ablation | TTTS MC twins not undergoing laser ablation | Abnormal cord insertion (velamentous and marginal) | 10/11 – low risk of bias | Strategies to address incomplete follow up was not applicable. |
| Slaghekke (2014)^47^ | Randomised control trial | 5 Countries | 74 placentas from TTTS MC twins undergoing the Solomon laser technique. 77 placentas from TTTS MC twins undergoing the selective laser technique | 41 placentas excluded due to placental maceration, 1 placenta excluded due to formalin fixation, 23 placentas excluded due to damage and 31 placentas lost | TTTS MC twins undergoing Solomon laser ablation | TTTS MC twins undergoing selective laser ablation | Proximate cord insertion and residual anastomoses present | Low risk of bias in all domains | \ |
| Starnes (2019)^48^ | Retrospective cohort | USA | 33 placentas from TTTS MC twins undergoing selective laser technique, 4 placentas from TRAP twins and 10 placentas with sIUGR | Incomplete data sets excluded from analysis | TTTS MC twins undergoing laser ablation | TRAP twins and twins with sIUGR | Abnormal cord insertion (velamentous and marginal) and residual anastomoses present | 9/11 – low risk of bias | Strategies to address confounding factors and incomplete follow up were not applicable |
| Van Den Wijngaard (2007)^15^ | Retrospective cohort | Netherlands | 12 placentas from TTTS MC twins undergoing laser and 14 placentas from non TTTS MC twins | 4 placentas excluded due to single or double intrauterine demise, 4 placentas excluded due to placental damage | TTTS MC twins undergoing laser ablation | Non TTTS MC twins | Residual anastomoses present | 8/11 – low risk of bias | Confounding factors not detailed. Strategies to address confounding factors and incomplete follow up were not applicable |
| Van Winden (2016)^49^ | Prospective cohort | USA | 64 placentas from TTTS MC twins undergoing laser and 91 placentas from TTTS and sIUGR twins | Intrauterine fetal demise, higher order multiples and placental distruption | TTTS MC twins undergoing laser ablation | TTTS and sIUGR MC twins undergoing laser ablation | Abnormal cord insertion (velamentous and marginal) | 9/11 – low risk of bias | Strategies to address confounding factors and incomplete follow up were not applicable |
| Wang (2022)^4^ | Retrospective cohort | China | 32 placentas from TTTS MC twins undergoing laser and 25 placentas from TTTS MC twins not undergoing laser | 111 placentas excluded as delivered at local hospital, 49 placentas excluded due to single or double intrauterine demise, 11 placentas excluded for placental damage | TTTS MC twins undergoing laser ablation | TTTS MC twins not undergoing laser ablation | Abnormal cord insertion (velamentous) and residual anastomoses present | 9/11 – low risk of bias | Strategies to address confounding factors and incomplete follow up were not applicable |
| Yonetani (2015)^20^ | Retrospective cohort | Japan | 30 placentas from TTTS MC twins. 20/30 undergoing laser (67%). 327 placentas from non TTTS MC twins | 5 cases excluded due to acardia, 1 case excluded due to acrania, 8 cases excluded due to intrauterine fetal demise or miscarriage and 6 cases excluded due to termination | TTTS MC twins undergoing laser ablation | Non TTTS MC twins | Abnormal cord insertion (velamentous and marginal) | 9/11 – low risk of bias | Strategies to address confounding factors and incomplete follow up were not applicable |
| Zhao (2013)^50^ | Retrospective case series | Netherlands | 252 placentas from TTTS MC twins undergoing laser | 148 placentas excluded as delivered at other centres, 11 placentas excluded due to single or double intrauterine fetal demise, 21 placentas excluded due to placental damage | TTTS MC twins undergoing laser ablation | \ | Proximate cord insertion and residual anastomoses present | 10/10 – low risk of bias | \ |

Only first author given for each study.

MC, monochorionic; sIUGR, selective intrauterine growth restriction; TAPS, twin anaemia-polycythaemia sequence; TRAP, twin reversed arterial perfusion; TTTS; twin-twin transfusion syndrome; USA, United States of America.
