# Supplementary material for: Placental architectural characteristics following laser ablation within monochorionic twins complicated by twin–twin transfusion syndrome: A systematic review and meta‐analysis of outcomes
Source: Acta Obstet Gynecol Scand. 2024 Jun 14;103(11):2130–46. doi: 10.1111/aogs.14891 (PMC11502458; doi:10.1111/aogs.14891)
Supplement: Supplementary file 3 — Table S3. [file AOGS-103-2130-s002.docx]

**Table S3**: Excluded studies and reason for exclusion.

| Author | Year | Title | Reason for the exclusion |
| --- | --- | --- | --- |
| Adegbite | 2003 | Perinatal outcome following amniotic septostomy in chronic TTTS is independent of placental angioarchitecture. | Not laser patients – amnioreduction and septostomy. |
| Akkermans | 2017 | What is the impact of placental tissue damage after laser surgery for twin-twin transfusion syndrome? A secondary analysis of the Solomon trial. | Wrong placental outcomes for this review. |
| Andrew | 2016 | Relationship of fetal complications in monochorionic diamniotic twin pregnancy with placental site cord insertion: A retrospective analysis of 3 | Does not separate laser and non-laser patients. |
| Atallah | 2017 | [Macroscopic description of placental vascular anastomoses after dye injection for the comprehension of monochorionic pregnancy complications]. | Does not separate laser and non-laser patients. 16 MCDA has laser, but not clear if all 9 in the TTTS cohort had laser. |
| Bajoria | 1998 | Chorionic place vascular anatomy determines the efficacy of amnioreduction therapy for twin-twin transfusion syndrome. | All patients had amnioreduction rather than laser. |
| Bajoria | 1998 | Vascular anatomy of monochorionic placenta in relation to discordant growth and amniotic fluid volume. | Patients not treated with laser. |
| Bajoria | 1999 | Outcome of twin pregnancies complicated by single intrauterine death in relation to vascular anatomy of the monochorionic placenta. | TTTS laser patients excluded from study. |
| Bajoria | 1995 | Angioarchitecture of monochorionic placentas in relation to the twin-twin transfusion syndrome. | TTTS laser patients excluded from study. |
| Bajoria | 1994 | Angioarchitecture of monochorionic placentas in relation to the twin-twin transfusion syndrome. | Duplicate. |
| Baschat | 2023 | Phenotype based staging of disease severity in twin-to-twin transfusion syndrome (TTTS). | Placental findings at laser, rather than at postnatal placental examination. |
| Becker | 1963 | Twin-to-twin transfusion syndrome. | Wrong study design. |
| Bendon | 1995 | Twin transfusion: Pathological studies of the monochorionic placenta in liveborn twins and of the perinatal autopsy in monochorionic twin pairs. | Monochorionic twins – does not specify TTTS. |
| Benirschke | 1995 | The biology of the twinning process: how placentation influences outcome. | Background article. |
| Benirschke | 2011 | Intrauterine growth restriction in twin-twin transfusion syndrome treated with laser surgery. | Abstract poster – insufficient information. |
| Benirschke | 2007 | Questions raised by placentas of laser interrupted TTTS. | Wrong study design. |
| Bermudez | 2002 | Twin-twin transfusion syndrome with only superficial placental anastomoses: endoscopic and pathological evidence. | Case report. |
| Bleisch | 1965 | Placental circulation of human twins: constant arterial anastomoses in monochorionic placentas. | No specific data for TTTS patients. |
| Brown | 1988 | Twin-twin transfusion syndrome sonographic findings. | Not documented about whether cohort underwent laser or not. |
| Bugerenko | 2021 | Twin anaemic polycythemia sequence after fetoscopy: Predictors of development. | Wrong population. |
| Bugerenko | 2020 | Long-term outcomes of laser coagulation of feto-fetal anastomoses in monochorionic twins. | Wong outcomes assessed. |
| Bugerenko | 2019 | Placental angioarchitecture in monochorionic twin pregnancies with twin-twin transfusion syndrome. Perinatal outcomes. | Anastomoses were detected during laser, not postnatally. |
| Cambiaso | 2016 | Discordance of cord insertions as a predictor of discordant fetal growth in monochorionic twins. | Not specific to TTTS patients. |
| Can | 2022 | Intertwin membrane cord insertion in dichorionic twin pregnancy: The description and comparison with other umbilical cord insertion types. | Not specific to TTTS patients. |
| Chan | 2010 | Incidence and clinicopathologic correlation of fetal vessel thrombosis in mono- and dichorionic twin placentas. | Unable to determine the number of TTTS patients that underwent laser. |
| Chang | 2011 | The relationship of umbilical venous volume flow, birthweight and placental share in monochorionic twin pregnancies with and without selective intrauterine growth restriction. | Not specific to TTTS laser patients. |
| Chang | 2022 | Effect of fetoscopic laser photocoagulation on fetal growth and placental perfusion in twin-twin transfusion syndrome. | Wrong outcomes reported for review. |
| Chang | 2015 | Fetoscopic laser coagulation of intertwin anastomoses reduces discordant placental autophagic activities in discordant twin growth. | Wrong outcomes for this review. |
| Chu | 2013 | Placental endoglin levels in diamniotic-monochorionic twin gestations: correlation with clinical and placental characteristics. | Excluded TTTS laser patients. |
| Costa-castro | 2013 | Velamentous cord insertion in monochorionic twins: Does it matter? | Does not elude as to whether TTTS group has laser or not. |
| Couck | 2019 | The assessment of placental sharing using X-ray angiogram versus digital photograph: A prospective study. | Unable to separate TTTS and MC twins from the whole cohort. |
| Couck | 2020 | The vascular equator in monochorionic twin placentas. | No specific data for TTTS patients. |
| Couck | 2016 | The placenta in twin-to-twin transfusion syndrome and twin anemic polycythemia sequence. | Background article. |
| Couck | 2018 | Does site of cord insertion increase risk of adverse outcome, twin-to-twin transfusion syndrome and discordant growth in monochorionic twin pregnancy? | Does not separate patients with laser and non-laser in TTTS cohort. |
| Couck | 2022 | The association between vein-to-vein anastomoses and birth weight discordance in relation to placental sharing in monochorionic twin placentas. | Excludes TTTS patients. |
| Danon | 2014 | Discordant placental mRNA expression in complicated monochorionic twins. | Wrong study design. |
| De Paepe | 2010 | Placental markers of twin-to-twin transfusion syndrome in diamniotic-monochorionic twins: A morphometric analysis of deep artery-to-vein anastomoses. | Laser treated TTTS patients excluded from analysis. |
| De Paepe | 2017 | Redness discordance in monochronic twin placentas: Correlation with clinical and placental findings. | Excludes TTTS placentas. |
| De Paepe | 2009 | The placental signature of twin-to-twin transfusion syndrome. | Wrong publication type. |
| De Paepe | 2015 | Examination of the twin placenta. | Background article. |
| De Paepe | 2002 | Demonstration of placental vascular anatomy in monochorionic twin gestations. | Unclear if TTTS cohort has laser or not. |
| De Paepe | 2013 | What and why the pathologist should know about twin-to-twin transfusion syndrome. | Background article. |
| De Villiers | 2012 | Arterio-arterial vascular anastomoses in monochorionic placenta with and without twin-twin transfusion syndrome. | Laser treated TTTS patients were excluded. |
| De Villiers | 2015 | Correlation between veno-venous anastomoses, TTTS and perinatal mortality in monochorionic twin pregnancies. | Laser treated TTTS patients were excluded. |
| De Vries | 2006 | Colour oscillations in arterioaterial anastomoses reflect natural differences in donor and recipient oxygenation and hematocrit. | Wrong study design. |
| Degenhardt | 2017 | Short-time impact of laser ablation of placental anastomoses on myocardial function in monochorionic twins with twin-to-twin transfusion syndrome. | Wrong outcomes reported for review. |
| Delabaere | 2023 | Fetal doppler in monochorionic pregnancies complicated by twin-to-twin transfusion syndrome and selective in utero growth restriction. | Wrong outcomes reported for review. |
| Denbow | 1998 | Colour doppler energy isonation of placental vasculature in monochorionic twins: absent arterio-arterial anastomoses in association with twin-twin transfusion syndrome. | Unclear and limited information on TTTS pregnancies. |
| Denbow | 2000 | Placental angioarchitecture in monochorionic twin pregnancies: relationship to fetal growth, fetofetal transfusion syndrome, and pregnancy outcome. | Cases of TTTS were not managed with laser. |
| Donepudi | 2016 | Recurrent twin-twin transfusion syndrome (rTTTS) and twin anemia polycythemia sequence (TAPS) after fetoscopic laser surgery (FLS). | Post abstract, groups TTTS and TAPS together. |
| Emery | 2016 | Histological appearance of placental solomonization in the treatment of twin-twin transfusion syndrome. | Wrong publication type. |
| Ferriman | 2018 | Twin pregnancy. | Background article. |
| Fichera | 2005 | Antenatal detection of arterio-arterial anastomoses by Doppler placental assessment in monochorionic twin pregnancies. | Does not elude to the number of patients that underwent laser within the TTTS group. |
| Fries | 1993 | The role of velamentous cord insertion in the etiology of twin-twin transfusion syndrome. | Laser not performed in TTTS cohort. |
| Galea | 2008 | The placenta contributes to activation of the renin angiotensin system in twin-twin transfusion syndrome. | Cases of laser were excluded. |
| Galea | 2005 | Insights into the pathophysiology of twin-twin transfusion syndrome. | Background article. |
| Gandhi | 2011 | Treatment of twin-twin transfusion syndrome with proximate umbilical cord insertion. | Wrong study design. |
| Gratacos | 2012 | A systematic approach to the differential diagnosis and management of the complications of monochorionic twin pregnancies. | Wrong study design. |
| Groene | 2022 | Neonatal management and outcome in complicated monochorionic twins: what have we learned in the past decade and what should you know? | Background article. |
| Groene | 2022 | Impact of placental sharing and large bidirectional anastomoses on birthweight discordance in monochorionic twins: a retrospective cohort study in 449 cases. | Wrong population. |
| Guilherme | 2008 | Zygosity and chronicity in the prognosis of triplet pregnancies: contribution of microsatellites. | Wrong population. |
| Gulati | 2019 | Classifying causes of in utero and neonatal death in twin pregnancies from postmortem examinations undertaken in a tertiary hospital over a 4-year period. | Abstract with insufficient information. |
| Hack | 2008 | Placental characteristics of monochorionic diamniotic twin pregnancies in relation to perinatal outcome. | Only 5 out of 30 TTTS patients had laser. |
| Haslik | 2021 | In vitro stimulation of acute feto-fetal transfusion in case of single intrauterine fetal death in monochorionic twins. | Wrong study design. |
| Haslik | 2019 | Acute feto-fetal transfusion simulation in monochorionic diamniotic twins. | Wrong study design. |
| Hecher | 2020 | Complicated monochorionic twin pregnancies. | Foreign language. Unable to digitally translate. |
| Hecher | 2008 | Monochorionicity: risks and intrauterine interventions. | Wrong study design. |
| Huang | 2001 | Velamentous cord insertion as a prognostic factor for twin gestations. | Wrong publication type. |
| Hubinont | 2015 | Anomalies of the placenta and umbilical cord in twin gestations. | Background article. |
| Jahanfar | 2018 | Placental pathology findings and birth weight discordance. | Not specific to TTTS patients. |
| Jelin | 2011 | Guide wire assisted catheterization and colored dye injection for vascular mapping of monochorionic twin placentas. | Wrong study design. |
| Joern | 1999 | Antenatal visualization of vascular anastomoses in monochorionic twins using color Doppler sonography: the protective function of thee anastomoses and the phenomenon of interference beating. | Wrong study design. |
| Jones | 1996 | Twin transfusion syndrome: reassessment of ultrasound diagnosis. | Wrong outcome. |
| Kalafat | 2018 | Significance of placental cord insertion site in twin pregnancy. | Not clear if TTTS patients were laser or non-laser. |
| Kalafat | 2018 | Placental cord insertion as a predictive marker for complications in multiple pregnancy. | Duplicate. |
| Kaneti | 2013 | Vascular network features, excluding anastomotic communications, account for birthweight discordance in monochorionic TTTS placentas. | Poster abstract with insufficient information. |
| Karpf | 2019 | Placenta in twin pregnancy. | Wrong study design. |
| Kent | 2011 | Placental cord insertion and birthweight discordance in twin pregnancies: results of the national prospective ESPRit Study. | Does not allude to the 13 TTTS patients whether they had laser or not. |
| Kim | 2020 | 764: Pathologic findings of placenta parenchyma in twin-to-twin transfusion syndrome differ according to stage. | Only includes TTTS without laser. |
| Konno | 2023 | Placental recruitment after spontaneous single fetal demise in monochorionic diamniotic twin pregnancies. | Wrong study design. |
| Konno | 2022 | Effect of superficial anastomoses on circulatory dynamics in twin-twin transfusion syndrome. | Wrong outcome reported. |
| Lacunza | 2015 | Placenta angio-architecture and origin of monochorial pathology. | Wrong population. |
| Lanna | 2015 | Colour-dye injection of monochorionic placentas and correlation with pregnancy complications. | Excludes laser patients. |
| Lee | 2021 | Clinical significance of velamentous cord insertion prenatally diagnosed in twin pregnancy. | Does not sperate TTTS patient with and without laser. |
| Lewi | 2010 | Monochorionic diamniotic twin pregnancies pregnancy outcome, risk stratification and lessons learnt from placental examination. | Background article. |
| Lewi | 2021 | Monochorionic twin placentas: complications of the shared circulation. | Wrong study design. |
| Lewi | 2022 | What fetal medicine specialists should know about the monochorionic placenta. | Background article. |
| Lewi | 2013 | The vascular anastomoses in monochorionic twin pregnancies and their clinical consequences. | Background article |
| Lia | 2009 | Placental laser surgery for severe previable feto-fetal transfusion syndrome in triplet gestation. | Wrong outcome. |
| Lipa | 2020 | Vascular anastomoses in intrauterine growth in monochorionic twins. | Could not clearly determine TTTS patients from total MC cohort. |
| Lopriore | 2014 | Acute peripartum twin-twin transfusion syndrome: Incidence, risk factors, placental characteristics and neonatal outcome. | Unable to determine how many has laser. |
| Lopriore | 2007 | Residual anastomoses after fetoscopic laser surgery in twin-to-twin transfusion syndrome: frequency, associated risks and outcome. | Data duplicated in 2009 study. |
| Lopriore | / | Placental characteristics in growth-discordant monochorionic twins: A matches case-control study. | Wrong population. |
| Lopriore | 2011 | Accurate and simple evaluation of vascular anastomoses in monochorionic placenta using colored dye. | Wrong study design. |
| Machin | 1996 | Correlations of placental vascular anatomy and clinical outcomes in 69 monochorionic twin pregnancies. | Data not specific to TTTS patients. |
| Machin | 2001 | The monochorionic twin placenta in vivo is not a black box. | Wrong study design. |
| Machin | 1996 | Zygosity and placental anatomy in 15 consecutive sets of spontaneously conceived triplets. | Wrong study design. |
| Machin | 2000 | Doppler sonographic demonstration of arterio-venous anastomosis in monochorionic twin gestation. | Wrong study design. |
| Miller | 2021 | Twin to twin transfusion syndrome. | Background article. |
| Morine | 2008 | Vascular endothelial growth factor in monochorionic twins with twin-twin transfusion syndrome. | None of the TTTS cohort underwent laser. |
| Murakoshi | 2011 | How to improve and shorten the learning curve of fetoscopic laser surgery for Twin-Twin transfusion syndrome. | Wrong publication type. |
| Murata | 2014 | The prevalence and clinical features of twin-twin transfusion syndrome with onset during the third trimester. | Wrong outcome. |
| Mustafa | 2022 | Placental characteristics in fetuses with fetal growth restriction undergoing laser ablation for twin-to-twin transfusion syndrome. | Unable to determine if anastomoses were detected postnatally on placental injection studies, the number of anastomoses are very high and imply were detected during laser. |
| Mustafa | 2022 | Proximate cord insertion in twin-to-twin transfusion syndrome: case series and PRISMA compliant systematic review and meta-analysis. | Wrong publication type. |
| Nakayama | 1996 | Monochorionic placenta and CNS disorder. Significance of anastomosis vessel on the placenta. | Unable to acquire full text. |
| Nikkels | 2008 | Pathology of twin placentas with special attention to monochorionic twin placentas. | Background article. |
| NTR1245 | 2008 | Solomon study. | Trial register. |
| Okamura | 1994 | Diagnostic use of cordocentesis in twin pregnancy. | Wrong outcome. |
| Pavlov | 2015 | Umbilical cord insertion type in twin-to-twin transfusion syndrome complicated diamniotic-monochorionic placentas. | Does not mention number of TTTS patients that had laser. |
| Pinho | 2023 | Velamentous cord insertion in monochorionic twin pregnancies: a step forward in screening for twin to twin transfusion syndrome and birthweight discordance? | Abnormal cord insertion only assessed antenatally. Unable to separate TTTS patients. |
| Quintero | 2005 | Individual placental territories after selective laser photocoagulation of communicating vessels in twin-twin transfusion syndrome. | Wrong outcome. |
| Quintero | 2000 | The donor-recipient (D-R) score: In vivo endoscopic evidence to support the hypothesis of a net transfer of blood from donor to recipient in twin-twin transfusion syndrome. | Wrong outcome. |
| Rand | 2009 | Natural history and outcomes of monochorionic twin pregnancies in a large population-based study. | Wrong publication type. |
| Rand | 2009 | Placental predictors of adverse outcomes in monochorionic twins. | Does not say which TTTS placentas had laser or not. |
| Robertson | 1983 | Placental injection studies in twin gestation. | Wrong study design. |
| Saenz | 2022 | Placental anastomoses in monochorionic twin pregnancies: Study using vascular injection techniques and relationship with fetal complications. | Only includes patients with non-laser TTTS. |
| Sago | 2022 | Placental abnormalities in twin, congenital anomaly and fetal therapy. | Wrong study design. |
| Sakata | 2006 | Fetal circulation and placental blood flow in monochorionic twins. | Background article. |
| Sau | 2008 | Antenatal detection of arteriovenous anastomoses in monochorionic twin pregnancy. | No TTTS case had laser. |
| Schrey | 2011 | Targeted gene arrays across the severely discordant growth monochorionic twin placenta: Implications for angiogenesis and metabolic programming. | Non TTTS patients. |
| Sen | 2018 | Monochorionic twin pregnancies: laser surgery. | Background article. |
| Shanahan | 2020 | Placental anatomy and function in twin gestations. | Background article. |
| Shao | 2013 | [Research of placental vascular distribution and clinical outcome in monochorionic twins]. | 50% cohort underwent laser. |
| Shiro | 2020 | Placental histopathological features of fetoscopic laser photocoagulation for monoamniotic diamniotic twin pregnancies. | Wrong outcome. |
| Slaghekke | 2014 | Residual anastomoses after fetoscopic laser coagulation of the vascular equator versus selective coagulation for twin-to-twin transfusion syndrome. | Duplicate. |
| Slaghekke | 2013 | Fetoscopic lasercoagulation of the entire vascular equator for treatment of twin-to-twin transfusion syndrome. ‘Solomon study’. | Post abstract with insufficient information. |
| Spruijt | 2020 | Twin-twin transfusion syndrome in the era of fetoscopic laser surgery: antenatal management, neonatal outcome and beyond. | Wrong study design. |
| Starnes | 2014 | Clinical co-morbidities, ablative site placental calcification, and vascular remodelling in twin-to-twin transfusion syndrome with and without selective fetoscopic laser photocoagulation (SFLP) | Post abstract with insufficient information. |
| Sun | 2014 | [Pregnancy outcome after fetoscopic laser photocoagulation for twin-twin transfusion syndrome: experience of an emerging center in China]. | Unable to digitally translate. |
| Sun | 2020 | Non-invasive dynamic observation of placental vascular anastomoses in monochorionic twins; assessment using three-dimensional sonography combined with tomographic ultrasound imaging. | 50% had laser in TTTS cohort. |
| Suzuki | 2011 | Influence of umbilical cord abnormalities (velamentous/marginal cord insertion and nuchal cord) on the perinatal outcomes of the second twin after vaginal delivery of the first twin. | Does not specifically document cord insertion for TTTS patients. |
| Szkodziak | 2022 | Twin-to-twin transfusion syndrome in monochorionic, monoamniotic twin pregnancy with common umbilical cord insertion. | Wrong publication type. |
| Szylberg | 2018 | Differences in the expression of TLR-2, NOD2 and NF-kB in placenta between twins. | Does not separative laser and non-laser TTTS patients. |
| Tagawa | 2014 | Placental pathology in twin to twin transfusion syndrome twins after fetoscopic laser photocoagulation. | Conference abstract with insufficient information. |
| Taylor | 2000 | Doppler detection of arterio-arterial anastomoses in monochorionic twins: feasibility and clinical application. | Cannot elude if TTTS cohort had laser or not. |
| Taylor | 2000 | Identification of arterio-venous anastomoses in vivo in monochorionic twin pregnancies: Preliminary report. | Case series. |
| Tollenaar | 2017 | Preoperative anemia-polycythemia in twin-twin transfusion syndrome: Incidence, placental characteristics and neonatal outcome. | Anastomoses not assessed post-operatively. |
| Umur | 2003 | Monoamniotic versus diamniotic-monochorionic twin placentas: anastomoses and twin-twin transfusion syndrome. | Cannot separate TTTS data from overall MC data. |
| Umur | 2002 | Monochorionic twins and twin-twin transfusion syndrome: the protective role of arterio-arterial anastomoses. | Wrong study design. |
| Van Gemert | 2001 | Polyhydramnios and arterio-arterial placental anastomoses may beneficially affect monochorionic twin pregnancies. | Wrong population. |
| Van Gemert | 1998 | Placental anatomy, fetal demise and therapeutic intervention in monochorionic twins and the transfusion syndrome: New hypotheses. | Wrong study design. |
| Van Winden | 2015 | Placental characteristics of monochorionic gestations complicated by twin-twin transfusion syndrome (TTTS) with and without selective intrauterine growth restrictions (SIUGR). | Abstract. Full paper in included studies. |
| Vladareanu | 2022 | Key points and challenges in monochorionic twins. | Background article. |
| Wang | 2011 | A study of the association about vegf, slft-1, flt-1 in dichorionic twins discordance. | Unable to acquire full text. |
| Wang | 2021 | Effect of placental vascular distribution on residual anastomoses after fetoscopic laser surgery for twin to twin transfusion syndrome. | Unable to digitally translate. |
| Wang | 2022 | Comparison of placental characteristics of twin-twin transfusion syndrome with and without selective intrauterine growth restriction. | Excludes TTTS patients undergoing laser. |
| Wang | 2021 | Longer distance between umbilical cord insertions is associated with spontaneous twin anemia polycythemia sequence. | Excludes TTTS patients undergoing laser. |
| Wang | 2021 | Study of the placental characteristics and time of onset of twin-to-twin transfusion syndrome. | Excludes TTTS patients undergoing laser. |
| Wang | 2015 | [Placental characteristics of twin-to-twin transfusion syndrome]. | Unable to digitally translate. |
| Waszak | 2011 | Monochorionicity in twin pregnancy as a possible factor endangering fetal development. | Wrong outcome. |
| Wee | 2006 | Histomorphometric characterisation of shared and non-shared cotyledonary villous territories of monochorionic placentae in relation to pregnancy complications. | Does not inform how many TTTS patients underwent laser. |
| Wee | 2005 | Characterisation of deep arterio-venous anastomoses within monochorionic placentae by vascular casting. | Only one TTTS patient. |
| Wee | 2003 | Transmitted arterio-arterial anastomosis waveforms causing cyclically intermittent absent/reversed end-diastolic umbilical artery flow in monochorionic twins. | Does not specifically describe TTTS patients. |
| Wee | 2004 | Reversal of twin-twin transfusion syndrome: frequency, vascular anatomy, associated anomalies and outcome. | Only documents reversal of TTTS in 5 out of 90 patients. Does not describe the full cohort. |
| Xueju | 2015 | A new indicator representing the efficiency of vascular anastomoses in monochorionic twin placentas. | Poster abstract with insufficient information. |
| Xueju | 2015 | Placental characteristics in spontaneous twin anaemia-polycythemia sequence. | Post abstract with insufficient information. |
| Yamamoto | 2021 | Prevalence of extraplacental anastomoses in monochorionic twin pregnancies. | Unable to extract fata specifically for TTTS patients. |
| Yang | 2011 | [Expression of HIF-alpha and its target gene in monochorionic twin placentas with twin-twin transfusion syndrome]. | Unable to digitally translate. |
| Zhao | 2016 | Histologic chorioamnionitis and funisitis after laser surgery for twin-twin transfusion syndrome. | Wrong outcome. |
| Zhao | 2014 | Monochorionic twin placentas: injection technique and analysis. | Background article. |
| Zhao | 2016 | Comparison between monochorionic and dichorionic placentas with special attention to vascular anastomoses and placental share. | Not TTTS specific. |
| Zhao | 2015 | ‘Superficial’ anastomoses in monochorionic placentas are not always superficial. | Patients not treated with laser were included. |
| Zhao | 2013 | Prevalence, size, number and localization of vascular anastomoses in monochorionic placentas. | Excludes laser TTTS patients. |
| Zhao | 2015 | Veno-venous anastomoses in twin-twin transfusion syndrome: a multicenter study. | Excludes laser TTTS patients |
| Zhao | 2014 | The role of veno-venous anastomoses in twin-twin transfusion syndrome. | Excludes laser TTTS patients. |
| Zhao | 2015 | Monochorionic placentas with proximate umbilical cord insertions: definition, prevalence and angio-architecture. | Excludes laser TTTS patients. |
